# A Bacterial Acetyltransferase Destroys Plant Microtubule Networks and Blocks Secretion

**DOI:** 10.1371/journal.ppat.1002523

**Published:** 2012-02-02

**Authors:** Amy Huei-Yi Lee, Brenden Hurley, Corinna Felsensteiner, Carmen Yea, Wenzislava Ckurshumova, Verena Bartetzko, Pauline W. Wang, Van Quach, Jennifer D. Lewis, Yulu C. Liu, Frederik Börnke, Stephane Angers, Andrew Wilde, David S. Guttman, Darrell Desveaux

**Affiliations:** 1 Department of Cell & Systems Biology, University of Toronto, Toronto, Ontario, Canada; 2 Centre for the Analysis of Genome Evolution & Function, University of Toronto, Toronto, Ontario, Canada; 3 Institut für Biologie, Lehrstuhl für Biochemie, Friedrich Alexander Universität Erlangen-Nürnberg, Germany; 4 Leslie Dan Faculty of Pharmacy, University of Toronto, Toronto, Ontario, Canada; 5 Department of Biochemistry, University of Toronto, Toronto, Ontario, Canada; 6 Department of Molecular Genetics, University of Toronto, Toronto, Ontario, Canada; Michigan State University, United States of America

## Abstract

The eukaryotic cytoskeleton is essential for structural support and intracellular transport, and is therefore a common target of animal pathogens. However, no phytopathogenic effector has yet been demonstrated to specifically target the plant cytoskeleton. Here we show that the *Pseudomonas syringae* type III secreted effector HopZ1a interacts with tubulin and polymerized microtubules. We demonstrate that HopZ1a is an acetyltransferase activated by the eukaryotic co-factor phytic acid. Activated HopZ1a acetylates itself and tubulin. The conserved autoacetylation site of the YopJ / HopZ superfamily, K289, plays a critical role in both the avirulence and virulence function of HopZ1a. Furthermore, HopZ1a requires its acetyltransferase activity to cause a dramatic decrease in *Arabidopsis thaliana* microtubule networks, disrupt the plant secretory pathway and suppress cell wall-mediated defense. Together, this study supports the hypothesis that HopZ1a promotes virulence through cytoskeletal and secretory disruption.

## Author Summary

Many bacterial pathogens disrupt key components of host physiology by injecting virulence proteins (or “effectors”) via a needle-like structure, called the type III secretion system, directly into eukaryotic cells. The YopJ / HopZ superfamily of type III secreted effector proteins is found in pathogens of both animals and plants providing an excellent opportunity to address how a family of type III secreted effectors can promote pathogenesis in hosts from two kingdoms. YopJ from the animal pathogen *Yersinia pestis* is an acetyltransferase that targets signaling components of innate immunity and prevents their activation. Here we show that HopZ1a, from the phytopathogen *Pseudomonas syringae* is an acetyltransferase that binds plant tubulin. Like YopJ, the eukaryotic cofactor phytic acid activates the acetyltransferase activity of HopZ1a. In addition, we demonstrate that activated HopZ1a can acetylate tubulin, a major constituent of the eukaryotic cytoskeleton. In plants, activated HopZ1a causes a dramatic destruction of microtubule networks, inhibits protein secretion, and ultimately suppresses cell wall-mediated defense. Our study emphasizes the functional diversification of this important type III effector family in plant and animal hosts using a conserved acetyltransferase activity.

## Introduction

The disruption of critical host cellular structures and processes is an important virulence tactic employed by bacterial pathogens of both plants and animals [1], [2]. Many Gram-negative bacterial pathogens accomplish this goal using the type III secretion system (T3SS) to inject virulence proteins known as type III secreted effectors (T3SEs) directly into the host cytosol [3]. One of the major virulence functions of phytopathogen T3SEs is to block host immune responses [4], [5]. These T3SEs employ a range of biochemical activities to modify host cell proteins and promote the infection process [6], [7]. However plants have evolved resistance (R) proteins that can recognize specific T3SE proteins to induce an effector-triggered immunity (ETI), which is often accompanied by localized cell death response called the hypersensitive response (HR) [8]–[10]. In some cases, plant resistance proteins directly bind T3SE proteins and induce ETI, but more typically, the actions of T3SEs on their host targets are monitored by plant resistance proteins as a trigger for ETI [11]. Therefore, T3SEs from phytopathogenic bacteria can act as either virulence or avirulence factors that promote bacterial growth or induce host immunity, respectively.

The YopJ superfamily of T3SEs is widely distributed in animal- and plant-pathogenic bacteria [12]. All members of this superfamily share a conserved catalytic triad consisting of the amino acids histidine, glutamic/aspartic acid, and cysteine that are characteristic of cysteine proteases [13], [14]. Despite sharing these catalytic residues, members of the YopJ superfamily display an array of biochemical activities. The archetypical member of this family, YopJ, displays de-sumoylating, de-ubiquitinating and acetyltransferase activity [13], [15]–[17]. The acetyltransferase activity of YopJ requires the eukaryotic cofactor phytic acid for full activation [18]. Phytic acid is also required for the full activation of AvrA, a YopJ homolog in *Salmonella* [18]. YopJ plays a crucial role in suppressing host immune responses, inhibiting cytokine production and inducing macrophage apoptosis [19], [20]. YopJ exerts its inhibitory activity by acetylating serine and threonine residues in the activation loop of mitogen-activated protein kinase (MAPK) superfamily, preventing their activation by phosphorylation and inhibiting downstream defense signaling pathways [15].

From plant pathogens, YopJ family members PopP2 from *Ralstonia solanacearum* displays acetyltransferase activity [21], AvrXv4 from *Xanthomonas campestris* pv. vesicatoria (*Xcv*) displays de-sumoylating activity [22] and members of the HopZ family from *Pseudomonas syringae* display protease activity [12]. PopP2 has been shown to autoacetylate residue Lys383, which is required for its avirulence activity in Arabidopsis [21]. Another YopJ homolog in *Xcv*, AvrBsT, has been shown to suppress AvrBs1-specific HR by interacting with SNF1-related kinase 1 (SnRK1), a putative regulator of sugar metabolism [23]. Lastly, HopZ1a and HopZ1b have been shown to interact with soybean GmHID1 (2-hydroxyisoflavanone dehydratase), which leads to suppression of daidzein biosynthesis. However, the direct mechanisms by which AvrBsT modify SnRK1 or HopZ1 modify GmHID1 are currently unknown as these effectors do not show proteolytic or acetyltransferase activity towards their targets [24], [25]. Thus, the enzymatic activities of many YopJ homologs in phytopathogens remain to be addressed.

The HopZ1 family of *P. syringae* T3SEs has diversified into three allelic forms (HopZ1a, HopZ1b and HopZ1c) with HopZ1a predicted to be most similar to the ancestral HopZ allele in *P. syringae* [12]. All members of the HopZ1 family contain a consensus myristoylation site (G2) required for membrane localization, suggesting that their host targets are membrane localized [26], [27]. In Arabidopsis, HopZ1a is recognized by the ZAR1 resistance protein [28]. This recognition requires the catalytic cysteine residue (C216) as well as the consensus myristoylation site (G2), suggesting that ZAR1 recognizes the membrane-localized enzymatic activity of HopZ1a [12], [26], [28]. In Arabidopsis plants lacking ZAR1, HopZ1a can promote *P. syringae* virulence suggesting that ZAR1 evolved to counter the ancestral virulence function of HopZ1a [28].

In this study, we show that HopZ1a, like YopJ, is an acetyltransferase that requires the eukaryotic co-factor, phytic acid, for full activation of its enzymatic activity. HopZ1a binds directly to tubulin and activated HopZ1a acetylates itself as well as tubulin. *In planta*, active HopZ1a causes a dramatic decrease in plant microtubule networks, inhibition of protein secretion and suppression of cell wall-mediated defense, supporting a novel mode-of-action for the YopJ superfamily.

## Results

### HopZ1a Binds to Tubulin and Microtubules Independent of the Conserved Catalytic C216 Residue

**Figure 1:**
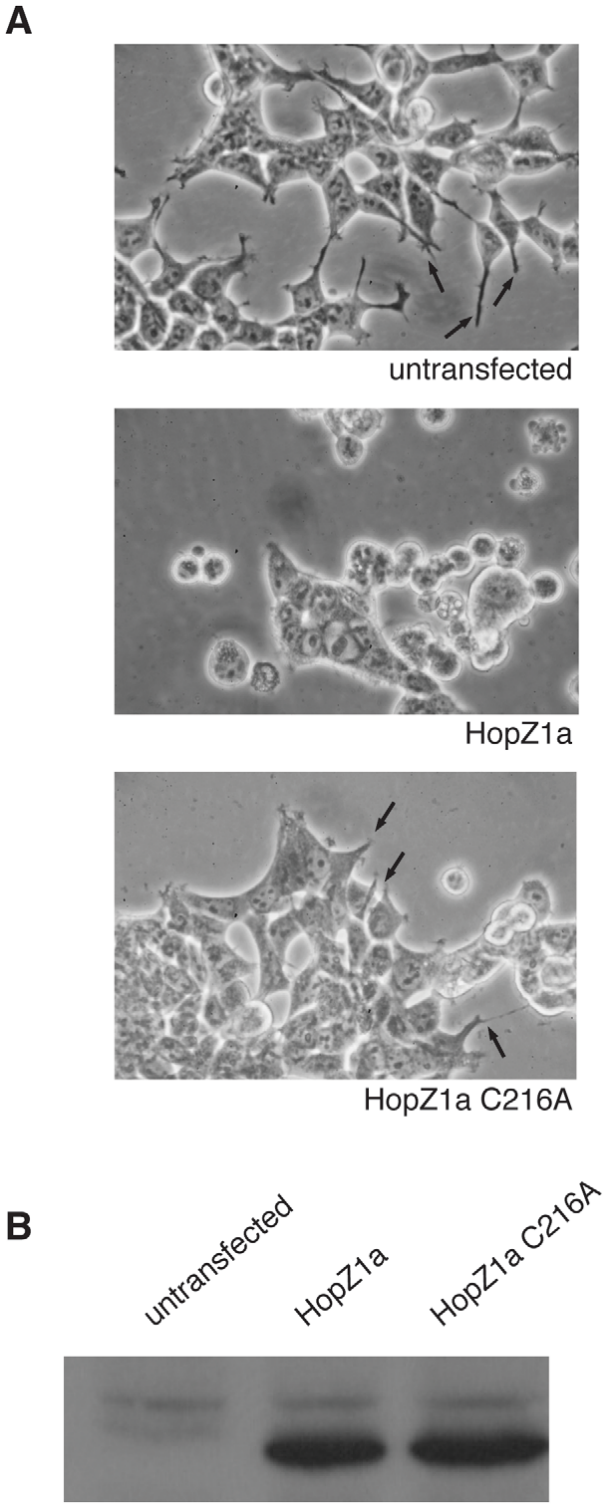
HEK293T Cells expressing HopZ1a have altered morphology. (**A**) HEK293T cells were transiently transfected with TAP-tagged HopZ1a and HopZ1a (C216A) for 24 h, followed by imaging of the transfected and untransfected cells. Arrows indicate cytoplasmic projections. (**B**) Immunoblot analysis of HEK293T cells transiently transfected with TAP-tagged HopZ1a and HopZ1a (C216A) with anti-HA antibody.

In an effort to identify conserved host targets of phytopathogen T3SEs, we utilized a heterologous *in vivo* screen whereby we expressed tandem-affinity-purification (TAP)-tagged phytopathogen T3SEs in human embryonic kidney (HEK293T) cells [29]. We hypothesized that those phytopathogen T3SEs that cause sick or lethal phenotypes in HEK293T cells are targeting conserved eukaryotic proteins. One T3SE identified from this screen was HopZ1a from *P. syringae* pv. *syringae* A2. HEK293T cells transiently expressing HopZ1a exhibited altered cell morphology compared to control HEK293T cells carrying the empty vector (Figure 1A), indicating a possible perturbation of the host cytoskeleton. The observed rounding and decrease in cytoplasmic projections of HEK293T cells expressing HopZ1a was dependent on the catalytic residue as it was not observed in cells expressing HopZ1a(C216A) (Figure 1).

We used TAP-tagged HopZ1a and liquid chromatography-tandem-mass spectrometry (LC-MS/MS) to recover eukaryotic proteins in complex with HopZ1a in HEK293T cells [29]. Tubulin was identified as an interacting protein based on its atypically large recovery and high sequence coverage (Table S1).

**Figure 2:**
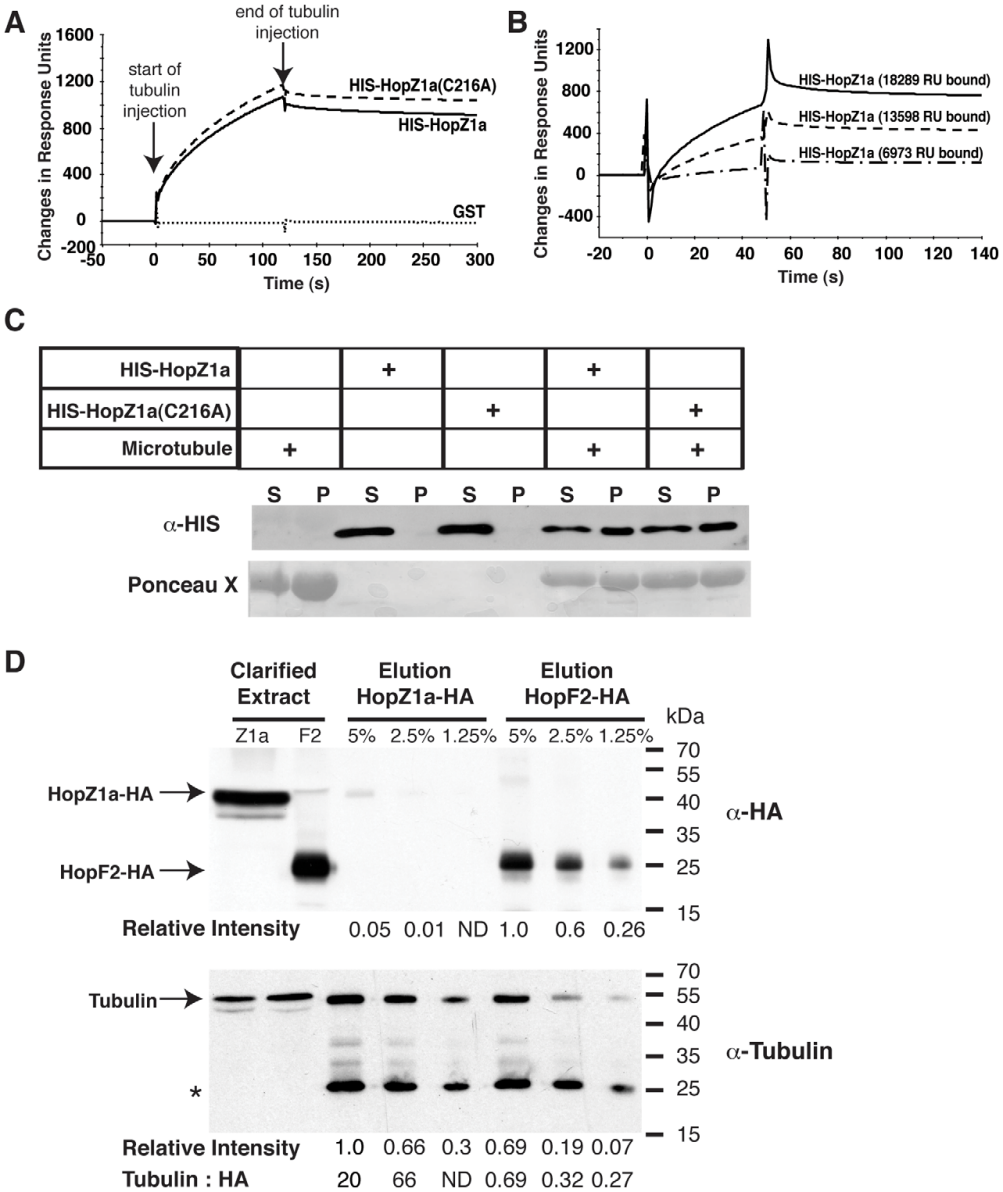
HopZ1a binds unassembled tubulin heterodimers and assembled tubulin polymers, the microtubules. (**A**) HIS-HopZ1a, HIS-HopZ1a(C216A) and GST were immobilized on the surface of a Biacore CM5 sensor chip at the following response units (RU), 9953 RU, 8784 RU and 3800 RU, respectively. 500 µg/ml of bovine brain tubulin was flowed across the recombinant HopZ1a, HopZ1a(C216A) and GST -bound surface, generating a RU difference of 1049 RU, 1150 RU and −12.2 RU, respectively. The start and end of tubulin injection are indicated by arrows. (**B**) 6973 RU, 13598 RU or 18289 RU of HIS-HopZ1a was immobilized on a Biacore CM5 sensor chip. 500 µg/ml of bovine brain tubulin was flowed across the recombinant HopZ1a-bound surfaces, generating a RU difference of 59 RU, 324 RU and 615 RU, respectively. (**C**) Immunoblot analysis of HIS-HopZ1a and HIS-HopZ1a(C216A) proteins in a microtubule co-sedimentation assay detected with rabbit α-HIS antibody. In the absence of microtubules, HIS-HopZ1a and HIS-HopZ1a(C216A) proteins were found only in the supernatant (S) fractions. In the presence of microtubules, HIS-HopZ1a and HIS-HopZ1a(C216A) proteins were found predominantly in the pellet (P) fractions. (**D**) HopZ1a binds to tubulin *in planta*. 1.5 ml of taxol-treated clarified extracts from transgenic Arabidopsis expressing HA-tagged HopZ1a(C216A) or HopF2 were bound to 30 µl of α-HA antibody-coated agarose beads. Proteins were eluted from the α-HA beads by boiling the beads in 100 µl of Laemmli sample buffer. 5 µl, 2.5 µl and 1.25 µl of the resulting eluates (corresponding to 5%, 2.5% and 1.25% of eluates) were subjected to immunoblots using α-HA antibodies (top panel) or α-tubulin antibodies (bottom panel). In the top panel 20 ul of HopZ1a clarified extract and 5 ul of HopF2 clarified extract was loaded whereas in the bottom panel 3 ul of each clarified extract was loaded, The band intensities of HopZ1a, HopF2, and tubulin bands were quantified by ImageJ and shown at the bottom of each panel. The maximum band intensity observed in each blot is arbitrarily set at 1 and all other band intensities are shown relative to that value. The ratios of tubulin:HopZ1a(C216A) and tubulin:HopF2 are indicated at the bottom of the figure. (*) indicates non-specific, cross-reactive bands.

To determine if purified HopZ1a directly interacts with tubulin heterodimers *in vitro* we used surface plasmon resonance technology to monitor real-time changes in the refractive index of bound HopZ1a associated with the presence of tubulin heterodimers. Tubulin is highly conserved across all eukaryotes (Arabidopsis and humans share ∼85% amino acid identity for both α-tubulin and β-tubulin). We therefore used commercially available purified bovine brain tubulin for this assay as high purity plant tubulin is not available. Both wild type HIS-HopZ1a and HIS-HopZ1a(C216A) catalytic mutant bound tubulin heterodimers, suggesting that HopZ1a has a tubulin-binding domain (TBD) that is independent of its enzymatic activity (Figure 2A). Additionally, tubulin heterodimers interacted with the purified HIS-HopZ1a proteins in a dosage-dependent manner (Figure 2B). The interaction between tubulin and HopZ1a was specific, as glutathione S-transferase (GST, Figures 2A, S1B and S1C) or bovine serum albumin (BSA, Figure S1A) did not bind to tubulin heterodimers. GST-HopZ1a also bound tubulin heterodimers, indicating that the fusion tags were not responsible for the interaction between tubulin and HopZ1a (Figures S1B and S1C). Lastly, HopZ1a bound to soybean tubulin (Figure S1C). However, due to the higher levels of impurities found in soybean tubulin, we conducted all subsequent experiments using bovine brain tubulin.

We next determined whether purified HopZ1a could bind to intact microtubules (as opposed to tubulin heterodimers) *in vitro* using a microtubule co-sedimentation assay [30] in which microtubule-binding proteins sediment with microtubules. We found that both purified HIS-HopZ1a and HIS-HopZ1a(C216A) co-sedimented with taxol-stabilized microtubules in the pellet fraction. In the absence of taxol-stabilized microtubules, HopZ1a and HopZ1a(C216A) remained in the supernatant (Figure 2C). Thus, as seen with the interaction between HopZ1a and tubulin heterodimers, HopZ1a also bound microtubules independent of its catalytic residue, C216. Moreover, this interaction is specific as the unrelated *P. syringae* type III effector, HopF2, remained predominantly in the supernatant fraction (Figure S2).

### HopZ1a Binds to Tubulin *in planta*

Given that HopZ1a binds to human and bovine tubulin, we next determined whether HopZ1a can bind to plant tubulin using co-immunoprecipitation (CoIP) on Arabidopsis transgenic plants expressing HopZ1a(C216A)-HA (Figure 2D). Despite a low level of HopZ1a(C216A)-HA immunoprecipitation (Figure 2D, top panel), a considerable amount of tubulin proteins were detected in the HopZ1a fractions (Figure 2D, bottom panel). Given that tubulin is an abundant protein that can be pulled down non-specifically in CoIP experiments, we used HopF2-HA transgenic plants as a negative control since HopF2 is not expected to bind to tubulin specifically (see Figure S2) and, like HopZ1a, is membrane localized via myristoylation. Although some tubulin was detected from the HopF2-HA eluted fractions, quantification of the band intensities of the α-tubulin and α-HA blots showed that the ratio of tubulin to HA-tagged effector was significantly lower than in the HopZ1a fractions. In fact, HopZ1a pulled down 30–200 times more tubulin than HopF2 demonstrating that HopZ1a binds to plant tubulin specifically.

### HopZ1a Is an Acetyltransferase Activated by the Eukaryotic Cofactor Phytic Acid

**Figure 3:**
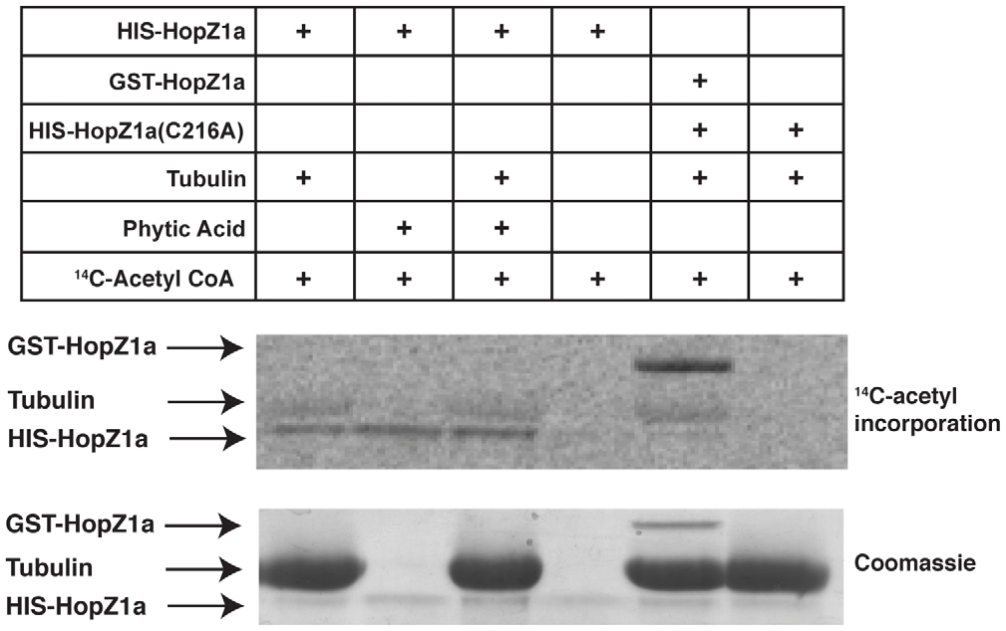
HopZ1a is an acetyltransferase activated by phytic acid and acetylates tubulin. Purified HIS-HopZ1a (∼42 kDa), GST-HopZ1a (∼68 kDa) and HIS-HopZ1a(C216A) (∼42 kDa) proteins were incubated with or without 10 µg of tubulin heterodimers (∼55 kDa) or 100 nM phytic acid in the presence of ^14^C-labeled acetyl-CoA for 1 hour at 30°C. The acetyltransferase activity of HopZ1a is activated by phytic acid. Active HopZ1a autoacetylates *in cis* and acetylates tubulin. All samples were separated by 12% SDS-PAGE and the ^14^C-incorporation was analyzed by Phosphorimager.

Since HopZ1a binds tubulin and microtubules, we next investigated whether active HopZ1a could modify tubulin. Based on the acetyltransferase activity of YopJ, we tested whether HopZ1a could perform a similar function via a ^14^C-labelled acetyl-coenzyme A (acetyl-CoA) transferase reaction [15], [31]. HIS-HopZ1a showed strong autoacetylation activity in the presence of tubulin and also weakly acetylated tubulin (Figures 3 and S3A, S3B and S3D). This activity was dependent on the conserved catalytic residue, C216, as the HIS-HopZ1a(C216A) mutant did not autoacetylate or acetylate tubulin (Figures 3 and S3D).

Phytic acid (also known as phytate, inositol hexakisphosphate and IP6) is an abundant eukaryotic cofactor that has been shown to be required for the full activation of number of YopJ family effectors [18]. Phytic acid is also a likely contaminant of purified bovine brain tubulin due to its abundance in this tissue [32]. Consequently, we asked whether HopZ1a was actually activated by phytic acid rather than tubulin. Acetylation assays showed that phytic acid activated the acetyltransferase activity of HopZ1a to the same extent as tubulin (Figure 3). Further, the removal of tubulin by Proteinase K treatment confirmed that HopZ1a's acetyltransferase activity was likely activated by contaminating phytic acid in the bovine tubulin (Figure S3C). Finally, mass spectrometry analysis of the bovine tubulin confirmed the presence of contaminating phytic acid (data not shown).

Using the molecular weight differences between GST-HopZ1a (∼70 kDa) and HIS-HopZ1a (∼41 kDa), we next investigated whether the autoacetylation on HopZ1a occurred in *cis* or in *trans*. Purified GST-HopZ1a showed a weak basal level of acetyltransferase activity in the absence of tubulin, possibly due to the GST tag stabilizing HopZ1a protein (Figure S3A). However, the acetyltransferase activity of GST-HopZ1a, like HIS-HopZ1a, was strongly activated by phytic acid (Figure S3A). GST-HopZ1a and HIS-HopZ1a migrate at different rates during electrophoresis; therefore, we could show that HopZ1a autoacetylated in *cis* since GST-HopZ1a strongly acetylated itself (in *cis*) and only weakly acetylated HIS-HopZ1a(C216A) (Figure 3). The reciprocal experiment showed that active HIS-HopZ1a strongly acetylated itself in *cis* and did not acetylate GST-HopZ1a(C216A) (Figure S3D). Taken together, our data show that (i) HopZ1a acetylates tubulin [33], HopZ1a requires eukaryotic co-factor phytic acid for full activation of its acetyltransferase activity and (iii) the HopZ1a autoacetylation activity occurs predominantly *in cis*.

### The Conserved Autoacetylation Site, lysine 289 (K289), Is Required for HopZ1a Avirulence and Virulence Functions

**Figure 4:**
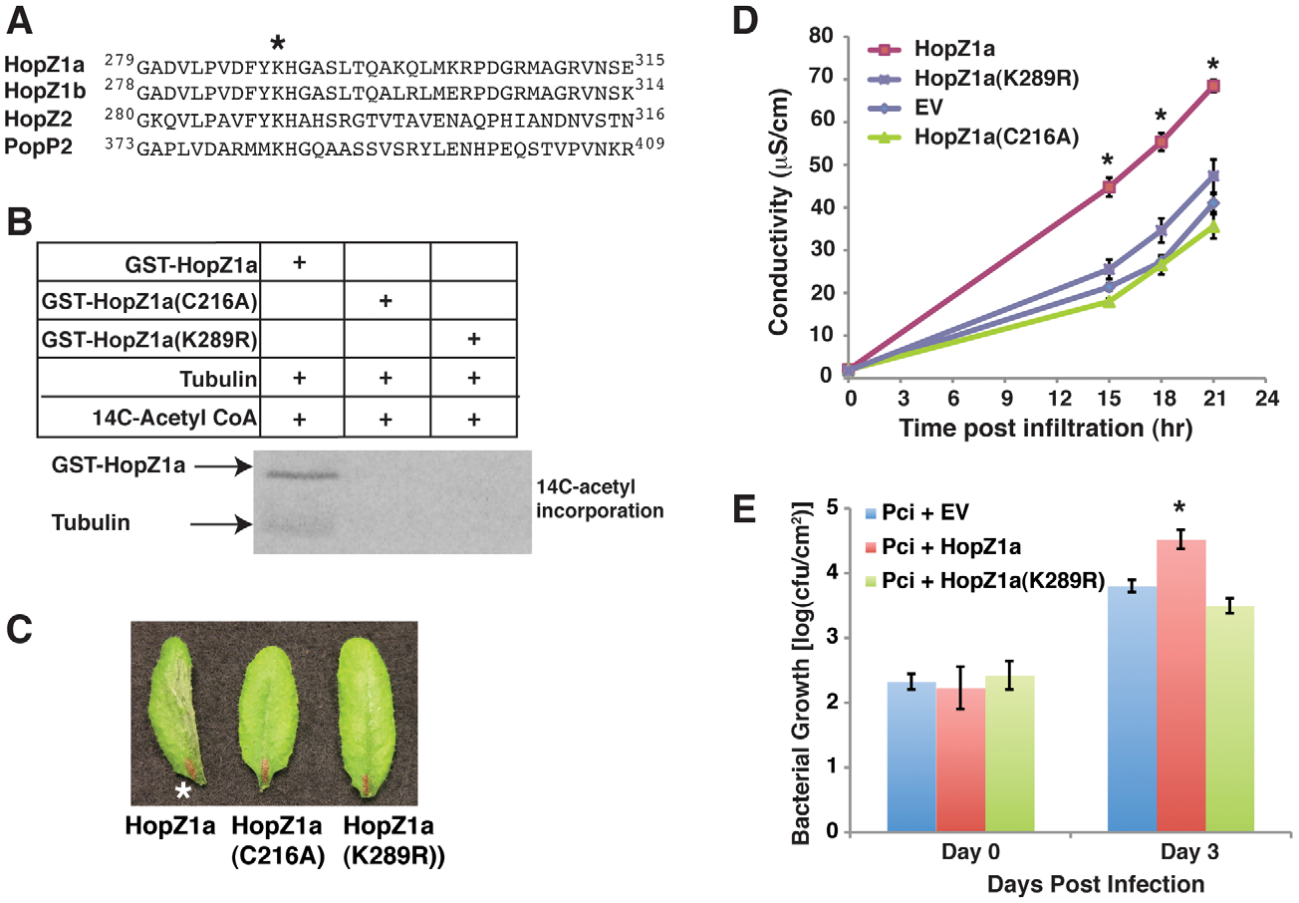
The autoacetylation site of HopZ1a, K289, is important for the avirulence and virulence function of HopZ1a. (**A**) The protein sequence of HopZ1a is aligned with HopZ1b, HopZ2 and PopP2 using Clustal W. The region flanking the conserved lysine residue is shown, with lysine 289 (in HopZ1a) indicated by a star. (**B**) Purified recombinant GST-HopZ1a, GST-HopZ1a(C216A) and GST-HopZ1a (K289R) proteins were incubated with tubulin heterodimers in the presence of ^14^C-labeled acetyl-CoA for 1 hour at 30°C. All samples were separated by 12% SDS-PAGE and the ^14^C-incorporation was analyzed by Phosphorimager. (**C**) Macroscopic HR of Arabidopsis Col-0 leaves infiltrated with 2×10^7^ CFU/ml of *Pto*DC3000 expressing *pUCP20-hopZ1a-HA* (HopZ1a WT), *pUCP20-hopZ1a(C216A)-HA* [HopZ1a (C216A)] or *pUCP20-hopZ1a(K289R)-HA* [HopZ1a(K289R)]. (*) indicate HR. (**D**) Quantification of HR by electrolyte leakage of Arabidopsis Col-0 leaf discs after infiltration with 5×10^7^ CFU/ml of *Pto*DC3000 expressing empty vector (EV), *pUCP20-hopZ1a-HA* (HopZ1a WT), *pUCP20-hopZ1a(C216A)-HA* [HopZ1a (C216A)], or *pUCP20-hopZ1a(K289R)-HA* [HopZ1a(K289R)]. Error bars represent standard error and (*) indicate statistically significant differences (2-tailed student t-test, p<0.01). The experiment was repeated twice with similar results. (**E**) *P. syringae* (*Pci*0788-9) growth assay in Arabidopsis. *Pci*0788-9 carrying *pUCP20-hopZ1a-HA* (HopZ1a WT) grew significantly better than *Pci*0788-9 carrying *pUCP20-hopZ1a(K289R)-HA* [HopZ1a(K289R)] or empty vector (EV) on day 3. The bacterial growth difference between HopZ1a WT and HopZ1a K289R or EV was statistically significant [as indicated by (*), 2-tailed student t-test, p<0.01]. Error bars represent standard error. Experiments were repeated three times and the data from one representative experiment is presented.

To understand the function of HopZ1a autoacetylation, we identified a potential autoacetylation site by aligning various effector proteins from the HopZ family (HopZ1a, HopZ1b and HopZ2) to their homolog in *R. solanacearum*, PopP2 (Figure 4A). Work by Tasset *et al* [21] has shown that K383 in PopP2 (which is homologous to K289 in HopZ1a) is autoacetylated and a mutation in K383 blocks R-protein (RRS1)- mediated recognition of PopP2. Thus, to address whether K289 is also required for HopZ1a function, we assayed the acetyltransferase activity of HopZ1a(K289R). In the presence of tubulin, GST-HopZ1a(K289R), like the catalytic-null GST-HopZ1a(C216A), did not display any acetyltransferase activity either towards itself or tubulin (Figure 4B). HopZ1a(K289R) tubulin-binding was similar to wild-type HopZ1a indicating that this mutation does not significantly affect the overall structure of the protein (Figure S4A). Additionally, in the absence of tubulin, GST-HopZ1a(K289R) did not show even the weak autoacetylation activity seen with the wild type GST-HopZ1a (Figure S4B). Therefore, the putative autoacetylation site, K289, is required for HopZ1a acetyltransferase activity.

Previous work has shown that HopZ1a induces a strong HR in *A. thaliana* ecotype Col-0 as a result of recognition by the ZAR1 resistance protein [12], [26], [28]. We monitored whether K289 of HopZ1a plays a role in host recognition by monitoring macroscopic HR symptoms (Figure 4C) as well as changes in conductivity due to HR-associated ion leakage (a quantitative measure for the HR; Figure 4D) of Arabidopsis leaves infiltrated with *P. syringae* pv. tomato DC3000 (*Pto*DC3000) expressing HopZ1a WT, HopZ1a(C216A), or HopZ1a(K289R). As previously shown, HopZ1a requires the catalytic C216 residue to induce a strong macroscopic HR and a corresponding increase in ion leakage in Arabidopsis ecotype Col-0 [12], [26], [28]. Interestingly, HopZ1a(K289R) phenocopied the catalytic-inactive HopZ1a(C216A) and did not elicit a macroscopic HR (Figure 4C). Furthermore, HopZ1a(K289R) induced conductivity similar to both *Pto*DC3000 carrying empty vector (EV) or the inactive HopZ1a(C216A) (Figure 4D). Thus, HopZ1a(K289R) plays a critical role in host recognition and the avirulence function of HopZ1a.

In the absence of the Arabidopsis resistance protein ZAR1, HopZ1a confers a virulence function to both *Pto*DC3000 and *P. syringae* pv. cilantro 0788-9 (*Pci*), in a catalytic (C216) dependent manner [28]. Like *Pto*DC3000, *Pci* strain does not carry an endogenous HopZ allele [12]. However, *Pci* carrying HopZ1a displayed a more significant growth increase compared to empty vector than what was observed in *Pto*DC3000 [28]. Therefore, we tested whether the K289 residue also played a role in HopZ1a virulence function by conducting *in planta* growth assays using *Pci* carrying the empty vector, wild type HopZ1a, and HopZ1a (K289R). In *zar1-1* plants, *Pci* expressing wild type HopZ1a promoted bacterial growth compared to *Pci* carrying the empty vector (Figure 4E), indicating that HopZ1a has a virulence function as previously shown [28]. However, the K289R mutation abolished HopZ1a virulence function (Figure 4E). Together, our data demonstrate that K289 contributes to HopZ1a: (i) acetyltransferase activity [33], avirulence function mediated by ZAR1 recognition, and (iii) virulence function.

### *P. syringae* Expressing Active HopZ1a Destroys Arabidopsis Microtubule Networks

Since HopZ1a bound tubulin *in vitro* and *in vivo*, we examined whether HopZ1a can induce structural changes to the host microtubule network. In order to minimize ETI-associated changes due to recognition of HopZ1a by ZAR1, we utilized a liquid assay where many features of ETI are suppressed [34], [35]. In brief, Arabidopsis seedlings expressing GFP-labeled microtubule markers were grown in liquid plant media and infected with *Pto*DC3000 expressing the empty vector [26], HopZ1a [26], HopZ1a(C216A) [26], or the unrelated *P. syringae* T3SE AvrRpt2 [36]. Since AvrRpt2 induces an HR in Arabidopsis within the same time frame as HopZ1a, it served as an additional control to ensure that any observed changes to the microtubule networks were HopZ1a-specific. We used confocal microscopy to observe changes in the 3-dimensional microtubule architecture of the epidermal layer at ∼16 hours post-infection with *Pto*DC3000.

**Figure 5:**
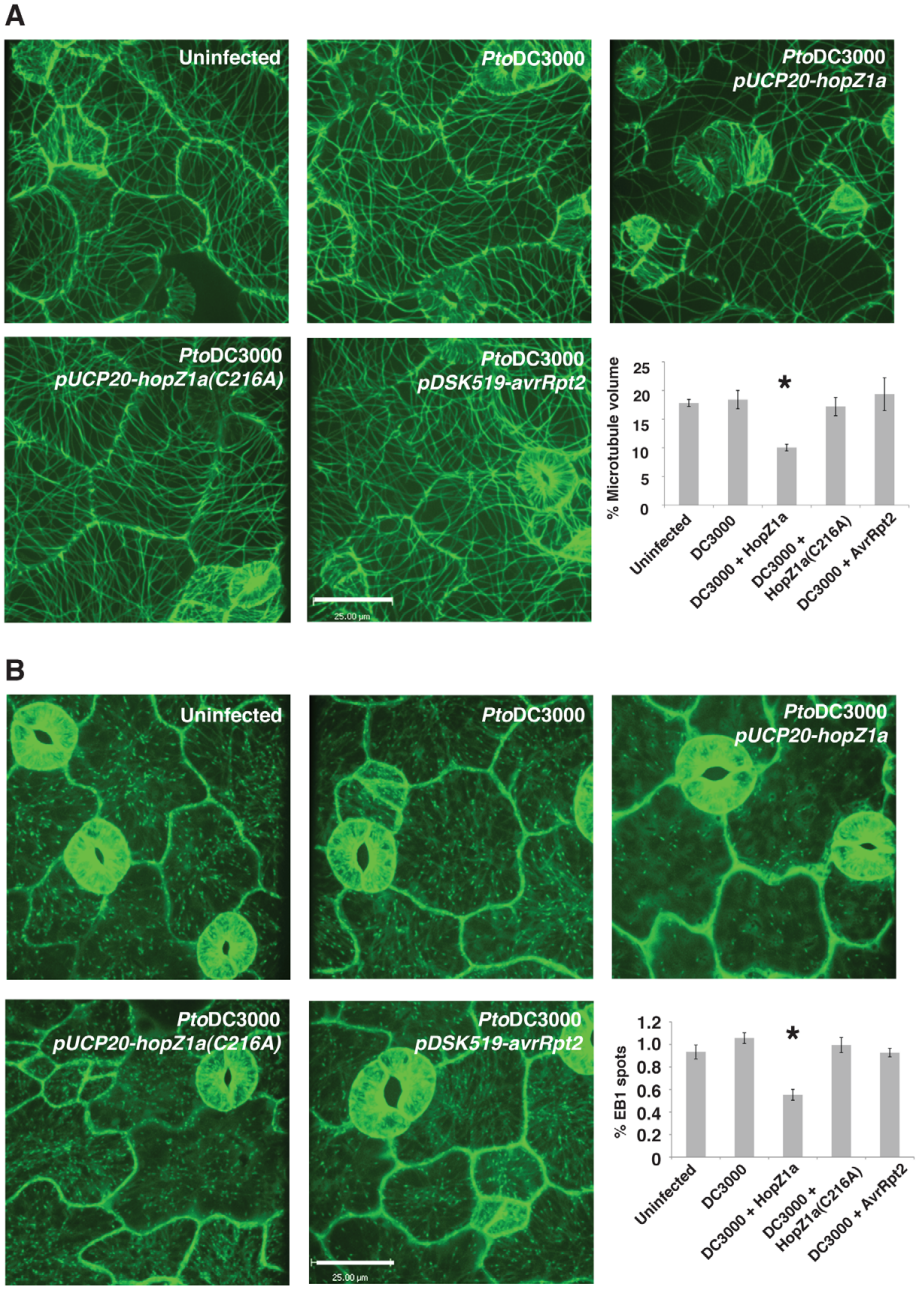
Effects of HopZ1a on the microtubule networks. Confocal microscopy images of five-day-old GFP-MAP4 (**A**) and GFP-AtEB1 (**B**) seedlings infected with *Pto*DC3000 expressing empty vector *pUCP20*, *pUCP20-hopZ1a-HA*, *pUCP20-hopZ1a(C216A)-HA*, or *pDSK519-avrRpt2* for ∼16 hours. Scale bar = 25 µm. (**A**) Quantification of the GFP fluorescence of GFP-MAP4 from 61 uninfected cells, 57 *Pto*DC3000-infected cells, 61 *Pto*DC3000(HopZ1a)-infected cells, 71 *Pto*DC3000(HopZ1aC216A)-infected cells and 67 *Pto*DC3000(AvrRpt2)-infected cells. (**B**) Quantification of the GFP fluorescence of GFP-AtEB1 from 37 uninfected cells, 82 *Pto*DC3000-infected cells, 127 *Pto*DC3000(HopZ1a)-infected cells, 90 *Pto*DC3000(HopZ1aC216A)-infected cells and 122 *Pto*DC3000(AvrRpt2)-infected cells. Error bars indicate standard error. [(*) indicate statistical significance. P = 0.05, Fisher's PLSD posthoc test.]

Two GFP-labeled microtubule markers were visualized: MAP4 [37], which associates with microtubules, and EB1 [38], which labels the growing ends of microtubules. At sixteen-hours post infection, *Pto*DC3000 carrying HopZ1a destroyed 50% of the cortical microtubule networks of Arabidopsis GFP-MAP4, whereas infection with *Pto*DC3000 carrying the empty vector, the HopZ1a(C216A) catalytic mutant, or AvrRpt2 induced no change in the cortical microtubule networks (Figure 5A). However, at later time points (∼22 hours post-infection), we observed microtubule destruction in all the treatments indicating that *Pto*DC3000 may alter the cytoskeleton at later time points (data not shown).

At sixteen hours post infection, *Pto*DC3000 carrying wild type HopZ1a also reduced the number of EB1 spots by 50%, while the other constructs showed no effect (Figure 5B). Note that the faint green haze in EB1 images were due to the fact that GFP-AtEB1 not only associates with the ends of microtubules, but also with the endomembrane system [38]. The microtubule-specific destruction in the presence of HopZ1a was not a result of general perturbation to the plant cytoskeleton, since *Pto*DC3000 carrying HopZ1a did not disrupt the actin cytoskeleton at the same time point (Figure S5). Thus, HopZ1a required its catalytic residue to specifically cause destruction of Arabidopsis microtubule networks.

### Microtubule Destruction Promotes *P. syringae* Virulence

**Figure 6:**
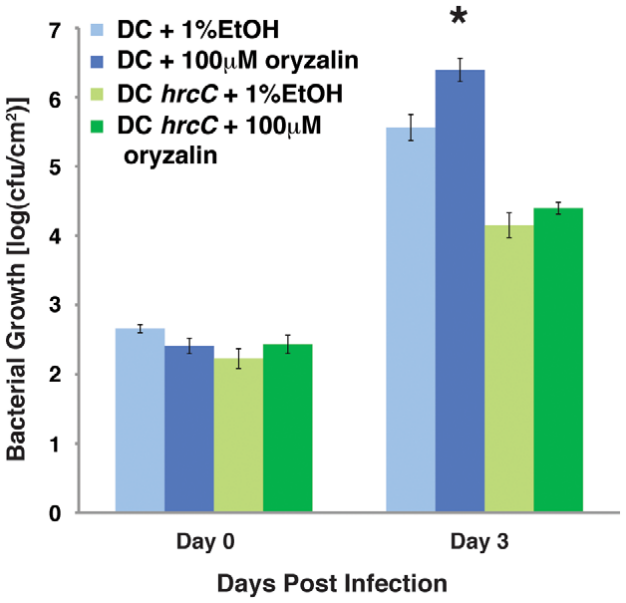
Microtubule destruction promotes *P. syringae* growth. *P. syringae* growth assay in Arabidopsis Col-0. In the presence of microtubule inhibitor, oryzalin, *Pto*DC3000 (DC) grew significantly better after three days, while *P. syringae* (DC *hrcC*) without a functional TTSS did not. The bacterial growth difference between DC in the presence or absence of oryzalin was statistically significant [as indicated by (*), 2-tailed student t-test, p = 0.008]. Experiments were repeated three times and the data from one representative experiment is presented.

As mentioned above, HopZ1a promotes *P. syringae* (*Pto*DC3000) growth in Arabidopsis plants lacking the ZAR1 R protein [28]. Given that the microtubule networks may play a crucial role in plant defense, we hypothesized that HopZ1a may promote the virulence of *P. syringae* via the destruction of microtubule networks. We used a pharmacological approach to determine whether the disruption of microtubules could account for HopZ1a-mediated enhanced virulence. When we co-infiltrated *Pto*DC3000 with the well-characterized disruptor of microtubules, oryzalin (100 µM in 1% ethanol), we observed significantly higher growth than *Pto*DC3000 co-infiltrated with 1% ethanol alone (Figure 6). A mutant lacking a functional T3SS, *Pto*DC3000 Δ*hrcC*, did not grow better in the presence of oryzalin, indicating that the virulence advantage of microtubule destruction requires a functional T3SS (Figure 6). Importantly, oryzalin did not provide a further virulence advantage to *Pto*DC3000 expressing HopZ1a, demonstrating that oryzalin can phenocopy HopZ1a virulence activity (Figure S6). Together, our results demonstrated that microtubule destruction promoted phytopathogen virulence.

### HopZ1a Interferes with the Plant Secretory Pathway

**Figure 7:**
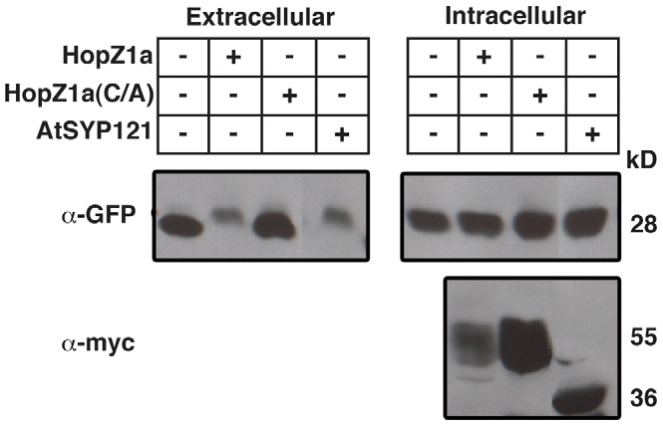
HopZ1a blocks the plant secretory pathway. Secretion assay in *N. benthaminana*. Extracellular fluid was isolated at 24 h post-inoculation from *N. benthamiana* expressing secGFP infiltrated with *Agrobacterium* carrying HopZ1a-myc [HopZ1a], HopZ1a(C216A)-myc [HopZ1a(C/A)], and AtSYP121-Sp2-myc [AtSYP121]. The expression of secGFP in the extracellular or intracellular fractions was analyzed by immunoblots using an anti-GFP serum; the expression of effectors was analyzed with anti-myc antibody. Transient expression of HopZ1a-myc, but not the catalytic mutant HopZ1a(C216A)-myc, reduced the accumulation of secGFP in the apoplastic fluid of *N. benthamiana*.

Microtubules play an important role in regulating vesicle trafficking and polarized secretion at the cortex [39], [40]. Given that plant microtubule networks were disrupted by catalytically active HopZ1a, we also investigated whether HopZ1a can block *in planta* secretion using a secreted green fluorescent protein (secGFP) assay. This assay has recently been used to demonstrate that the YopJ / HopZ family member, XopJ, can inhibit secretion *in planta* [41]. Normally, secGFP is secreted into the extracellular space (also known as the apoplast) of *Nicotiana benthamiana* leaves via a constitutive secretion pathway [41], [42]. However, accumulation of secGFP in apoplastic fluids from plants expressing wild type HopZ1a-myc was significantly reduced, demonstrating that HopZ1a blocked secretion to the same extent as the known secretion inhibitor, AtSYP121-SP2-myc (Figure 7). Furthermore, an unrelated T3SE, XopB, did not inhibit the secretion of secGFP to the apoplast (S. Sonnewald, personal communication), indicating that inhibition of secretion is not a general feature of type III effectors. The decreased accumulation of secGFP in the apoplastic fluids of plants expressing HopZ1a-myc was not due to decreased expression of secGFP or the induction of HR, as GFP was detected in the remaining tissues after apoplastic fluid extraction. The ability of HopZ1a to inhibit secretion was dependent upon the catalytic residue, C216, as secGFP was detected in the apoplastic fluids from plants expressing HopZ1a(C216A)-myc at similar levels to untransfected samples (Figure 7).

### HopZ1a Blocks Cell Wall-based Defense

Given that HopZ1a caused microtubule destruction, inhibited secretion, and the fact that plant microtubules are largely associated with the cell cortex [43], [44], we next investigated whether HopZ1a can interfere with cell wall-based defenses such as callose deposition. Callose, a key component of cell wall papillae, is composed of β-(1,3)-glucan polymers. These callose-containing papillae form important barriers at the sites of pathogen attack and are typically induced by the recognition of conserved pathogen- or microbe- associated molecular patterns (PAMPs/MAMPs) [45], [46]. However, *P. syringae* can counteract these cell wall-based defenses and other components of the PAMP-triggered immunity (PTI) by injecting virulence factors such as T3SEs into the plant [5], [47].

**Figure 8:**
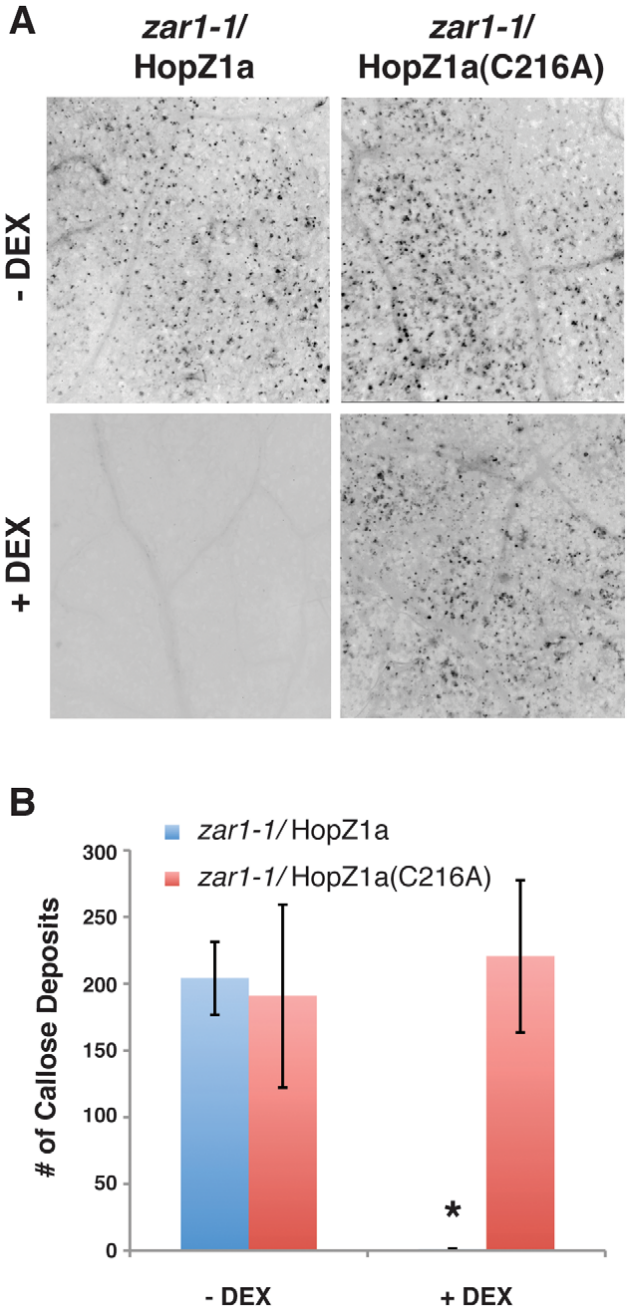
HopZ1a inhibits cell wall-based defense. (**A**) *zar1-1/Dex:hopZ1a* and *zar1-1/Dex:hopZ1a(C216A)* transgenic leaves were sprayed with water (−DEX) or 30 µM dexamethasone to induce HopZ1a protein expression (+DEX) for 24 h. Leaves were then syringe-infiltrated with 10 µM of flg22 for 24 h, followed by clearing and staining with 0.01% Aniline blue for callose. Expression of HopZ1a (+DEX), but not HopZ1a(C216A), suppressed flg22-induced callose deposition. (**B**) Quantification of callose depositions of 12 images per treatment. Error bars indicate standard error.

To determine whether HopZ1a can suppress cell wall-based defenses, we treated Arabidopsis leaves with a well-known PAMP, flg22, followed by staining with Aniline blue for callose (Figure 8). As mentioned above, HopZ1a triggers a strong HR due to the recognition by the ZAR1 R protein [28]. Therefore, transgenic plants expressing HopZ1a were generated in the *zar1-1* background. As expected, flg22 elicited strong callose deposition in the *zar1-1* plants (Figure S7). However, in *zar1-1* plants expressing HopZ1a flg22-induced callose deposition was suppressed demonstrating that HopZ1a blocked cell wall-defenses (Figure 8). Importantly, HopZ1a required the catalytic C216 residue to block flg22-induced callose deposition (Figure 8).

## Discussion

Bacterial pathogens use the type III secretion system to inject virulence proteins directly into the host cell. While we know the identity and general activity (e.g. suppression of defense signaling) of many phytopathogen T3SEs, relatively few have well-characterized enzymatic activities or host interactors. We used a heterologous screen to identify conserved host proteins that interact with phytopathogen T3SEs. We demonstrated that *P. syringae* T3SE, HopZ1a, is an acetyltransferase that interacts with tubulin. Furthermore, active HopZ1a alters microtubule networks, inhibits secretion and blocks cell wall-based defenses.

Plant microtubule networks are tightly associated with the cell membrane and this cortical association is important for maintaining the stability and organization of microtubule architecture in the plant cells [43], [44]. Unlike animal cells, plant microtubule arrays are non-centrosomal and nucleated by membrane-associated gamma-tubulin [48], [49]. A number of membrane-associated proteins, such as phospholipase D, provide additional tethering of microtubules to the membrane [50]. Given the importance of cortical association to plant microtubule stability and dynamics, it is not surprising that the membrane-localized HopZ1a can cause a dramatic destruction to the plant microtubule network [26].

We show that HopZ1a acetylates tubulin *in vitro*, however, this activity is notably weaker than the autoacetylation activity (Figures 3 and S3). It is possible that the *in vivo* microtubule context may be more readily acetylated by HopZ1a or that weak acetylation of tubulin *in vivo* may be sufficient to alter the assembly or disassembly dynamics of microtubules [51]. Recent work by Chu et al (2010) [52] has shown that acetylation on lysine 252 (K252) of beta-tubulin prevents incorporation of acetylated tubulin into the microtubule and alters microtubule polymerization, thus providing a potential mechanism by which HopZ1a alters plant microtubule networks. The acetylation of a subpopulation of tubulin by HopZ1a may be sufficient for lowering the critical concentration necessary for microtubule assembly, leading to the dramatic microtubule destruction that we observed. Alternatively, HopZ1a may cause microtubule destruction by acetylating as yet unidentified host protein(s), such as MAPKs, that are involved in regulating the stability of cortical microtubule networks [53]. Lastly, we cannot rule out that HopZ1a alters the microtubule networks as a consequence of ZAR1-mediated recognition. Experiments with *zar1*/GFP-MAP4 plants will address whether HopZ1a-mediated microtubule destruction is *ZAR1*-dependent.

Interestingly, the acetyltransferase activity of HopZ1a is activated by phytic acid (Figure 3), which plays an important role in defense signaling in addition to functioning as phosphate reserve in plants [54]. Phytic acid has been previously shown to activate the acetyltransferase activities of YopJ and AvrA, both highly divergent homologs of HopZ1a from animal pathogens [18]. Upon binding to phytic acid, AvrA undergoes a conformational change, which Mittal *et al* speculate leads to the allosteric activation of the acetyltransferase activity of AvrA [18]. Given that the catalytic inactive AvrA mutant also undergoes a similar conformational change upon binding to phytic acid, the phytic acid-binding site of AvrA is likely to be distant from the active site. It remains to be determined whether phytic acid induces similar conformational changes in HopZ1a and whether this putative conformational change is responsible for the activation of its enzymatic activity. However, the requirement for eukaryotic co-factor to fully activate HopZ1a's acetyltransferase activity suggests that this virulence protein is only ‘armed’ after injection into the host. This activation is reminiscent of the *P. syringae* T3SE AvrRpt2, which requires the eukaryotic cofactor cyclophilin to activate its cysteine protease activity, keeping this T3SE inactive until after delivery into the eukaryotic host cell [55], [56].

Recent work by Zhou *et al* [25] has identified an isoflavone biosynthesis enzyme GmHID1 as a target of HopZ1a and the closely related HopZ1b in soybean. Both HopZ1a and HopZ1b interact with the soybean GmHID1 protein *in vivo* and *in vitro*. However, Zhou *et al* did not observe any acetyltransferase activity of HopZ1 in the presence of GmHID1 and ^14^C-labeled acetyl CoA [25]. We speculate that the lack of acetyltransferase activity of HopZ1a is most likely due to the absence of the appropriate eukaryotic co-factor, or that while HopZ1 may bind to GmHID1, it is not a direct target of its enzymatic activity.

Previously we showed that the HopZ family has low-level *in vitro* protease activity using fluorescently-labeled casein as a generic substrate [12]. While our current confirmation of acetyltransferase activity is apparently at odds with these original findings, Mukherjee *et al.* [31] describe a “ping-pong” acetylation mechanism in which acetyltransferases and proteases use the same catalytic mechanism on different substrates. Consequently, it is possible that the acetyltransferase catalytic triad of the HopZ T3SEs could induce the weak fluorescence observed in our original assay, although the target of protease activity remains to be identified.

Upon activation by phytic acid, HopZ1a strongly autoacetylates in *cis*. Like its *R. solanacearum* homolog PopP2 [21], a mutation in the conserved lysine residue (K289 in HopZ1a) abrogates HopZ1a's autoacetylation activity and disrupts host recognition as evident by the lack of HR (Figures 3A–C). These results further support that it is the activity of HopZ1a that is recognized by the ZAR1 resistance protein in Arabidopsis [12], [26], [28]. In addition, the HopZ1a(K289R) mutant loses its virulence function and cannot promote bacterial growth in plants lacking the resistance protein ZAR1 (Figure 3D). These data indicate that the K289 autoacetylation site is conserved among members of the YopJ / HopZ superfamily and contributes to both virulence and avirulence functions.

Microtubules play an essential role in intracellular vesicle transport, structural support, cell division, developmental processes, and potentially host defense against fungal and oomycete pathogens [39], [57]–[60]. However, the role of microtubules in plant immunity against bacterial pathogens is unknown. Our data provide evidence that microtubules play an important role in defense against bacterial pathogens as the microtubule inhibitor oryzalin promotes *P. syringae* growth. However, the relatively weak growth advantage conferred to *Pto*DC3000 by oryzalin treatment may suggest that this pathovar possesses type III effectors that can target microtubules and/or possess activities that are functionally equivalent to microtubule destruction. Furthermore, the observation that oryzalin treatment did not promote *Pto*DC3000 Δ*hrcC* suggests that microtubule destruction is not sufficient to disrupt basal resistance/PTI and that microtubule destruction is just one component of the net virulence activity used by *Pto*DC3000 to promote *in planta* growth. We have also demonstrated that HopZ1a can block secretion, which may be partially as a result of microtubule destruction. Components of the secretory pathway have been demonstrated to play a role in resistance to *P. syringae* in Arabidopsis [61], [62], as well as in other pathosystems [63]. Most importantly, HopZ1a inhibits cell wall-based defenses by suppressing callose deposition in response to PAMP. Given that the cytoskeleton is important for trafficking of the flagellin receptor FLS2 [64], [65] and potentially the proper positioning of callose synthase, we speculate that a component of HopZ1a PTI-suppression is a consequence of microtubule destruction. Thus, by altering cortical microtubules with HopZ1a, *P. syringae* could affect intracellular transport and secretion thereby uncoupling defense signaling and cell wall-based defenses [45], [59], [60], [66].

## Materials and Methods

### Cloning

Promoter-less *hopZ1a* without the stop codon was PCR amplified from *P. syringae* pv. *syringae* A2 and cloned into the C-terminal TAP fusion vector [29]. Promoter-less *hopZ1a* was PCR amplified and cloned into the Gateway pENTR vector (Invitrogen, USA) using the BP reaction, followed by subcloning into the pDEST15 GST purification vector using the LR reaction. The HIS-HopZ1a and HIS-HopZ1a(C216A) purification constructs in pET14b (Novagen, USA) were described by Ma *et al.* [12]. All catalytic *hopZ1a* (C216A) mutants were generated using the QuikChange site-directed mutagenesis kit (Agilent Technologies, USA). To make *hopZ1a/zar1-1* transgenic plants, *hopZ1a* with an in-frame HA tag was PCR amplified and cloned into the dexamethasone-inducible pBD vector (a gift from Dr. Jeff Dangl, University of North Carolina, Chapel Hill, NC, USA).

### Tissue Culture and Transfection

Transient transfections of HEK293T cells were performed as previously described [29]. In brief, 5 µg of plasmid DNA and 5 µg of carrier DNA were added to 50 µl of 2.5 M CaCl_2_, 400 µl of sterile ddH_2_O and 500 µl of 2× HEPES-buffered saline (HBS) [50 mM HEPES pH 7, 10 mM KCl, 12 mM dextrose, 280 mM NaCl and 1.5 mM Na_2_PO_4_]. The calcium phosphate-DNA suspension was added drop-wise to 10 cm-plates of HEK293T cells. Cell culture media was changed 16 hours post transfection. Images of transfected and untransfected cells were taken 24 hours after transfection at 40× magnification, using Nikon Eclipse TS100 microscope and Canon DS6041 camera.

### Tandem Affinity Purification, Mass Spectrometry and Immunoblot

HEK293T cells expressing the TAP-tagged HopZ1a wild-type construct were used for tandem affinity purification and subsequent LC-MS/MS analysis as previously established [29]. HEK293T cells expressing HopZ1a-TAP or HopZ1a(C216A)-TAP were lysed in TAP-lysis buffer [0.1% Igepal CA 630, 10% glycerol, 50 mM HEPES; pH 8.0, 150 mM NaCl, 2 mM EDTA, 2 mM DTT, 10 mM NaF, 0.25 mM NaOVO3, 50 mM beta-glycerophosphate, and protease inhibitor cocktail (Sigma, USA)] for 15 min at 4°C. The cleared lysates were separated by 12% SDS-PAGE, transferred to nitrocellulose membrane and detected by anti-HA antibodies (Sigma, USA) via chemiluminescence (Amersham, USA).

### Purification of HopZ1a and HopZ1a(C216A) Proteins

The His-tagged HopZ1a and HopZ1a(C216A) proteins were overexpressed in *Escherichia coli* BL21-Gold (DE3) cells (Stratagene, USA) [12], purified using High Trap chelating columns (GE Healthcare, USA) preloaded with nickel, followed by size-exclusion chromatography using the AKTA FPLC with the Superdex S-200 column (GE Healthcare, USA). The GST-tagged HopZ1a and GST proteins were purified using glutathione Sepharose 4B column (GE Healthcare, USA).

### Surface Plasmon Resonance Binding Assay

CM5 research grade sensor chips were docked into the Biacore 3000 biosensor (GE Healthcare, USA) and preconditioned in HPS-EP running buffer [(0.01 M HEPES pH 7.4, 0.15 M NaCl, 3 mM EDTA, 0.005% Surfactant P20) (GE Healthcare, USA)] with 3 consecutive pulses of 10 ul each of 50 mM HCl, 50 mM NaOH, 0.5% SDS, and water at a flow rate of 100 ul/min. Injection of 200 mM 1-Ethyl-3-(3-dimethylaminopropyl) carbodiimide hydrochloride, (EDC) and 50 mM N-Hydroxysuccinimide (NHS) activated the CM5 sensor chip surface. 100 µg of purified recombinant HopZ1a, HopZ1a (C216A) and GST proteins were immobilized on CM5 chips in Biacore 3000 (GE Healthcare, USA) at a flow rate of 10 µl/min at 25°C by amine coupling using the Biacore Surface Preparation Wizard, followed by injection of 1 M Ethanolamine (GE Healthcare, USA) to block any remaining activated ester groups. Binding experiments were conducted at 25°C in HPS-EP running buffer in HPS-EP running buffer. 500 µg/ml of bovine brain tubulin or soybean tubulin (Cytoskeleton Inc., USA) was injected over the sensor chip surface BIAcore Quickinject protocol.

### Microtubule Sedimentation Assay

Taxol-stabilized microtubules were prepared as previously described [30]. In brief, 2.5 mg/ml of bovine brain tubulin (Cytoskeleton Inc., USA) was resuspended in 1× BRB80 (80 mM PIPES pH 6.8, 1 mM MgCl2, 1 mM EGTA) plus 1 mM DTT, 1 mM GTP and 5% DMSO, followed by 30 min incubation at 37°C. Polymerized microtubules were centrifuged at 55,000×*g* for 15 minutes at 25°C. The microtubule pellet was resuspended in warm 1× BRB80 with 10 µM Taxol to generate Taxol-stabilized microtubules. Purified recombinant HIS-HopZ1a, HIS-HopZ1a (C216A) and HIS-HopF proteins were pre-cleared of protein aggregates by centrifugation at 100,000×*g* for 10 minutes at 4°C. 1 µg each of pre-cleared HopZ1a, HopZ1a(C216A) and HopF2, in the presence or absence of 0.25 mg/ml of Taxol-stabilized microtubules, were incubated for 30 minutes at 25°C. Microtubules were recovered from the solution by centrifugation at 55,000×*g* for 15 minutes at 25°C. The resulting pellet (P) and supernatant (S) fractions were separated by SDS-PAGE, transferred to nitrocellulose membrane and detected with Ponceau X stain followed by α-His antibodies (Cell Signaling Technology, USA) via chemiluminescence (Amersham, USA).

### Co-immunoprecipitation of HopZ1a and Tubulin

HopZ1A (C216A)-HA and HopF2-HA transgenic Arabidopsis leaves were snap frozen in liquid nitrogen and then 1 g of tissue was homogenized in 2 ml of ice-cold extraction buffer composed of HEPES-KOH (pH 7.5), 50 mM KCl, 2 mM EDTA, 1 mM DTT, 0.2% Triton-X 100, 0.1 mg/ml dextran and 1∶100 (v/v) plant protease inhibitor (Sigma #P9599, USA). Homogenates were centrifuged at 4°C and 10,000×g for 10 min, and the supernatants reserved as clarified extract. Final concentrations of 1 mM GTP and 10 µM taxol were added to clarified extracts, followed by incubation at room temperature for 30 min to stabilize microtubules. Subsequently, 1.5 ml of stabilized extracts were subjected to co-immunoprecipitation by the addition of 30 µl α-HA IgG antibodies conjugated to agarose beads (Sigma; A2095) and incubated overnight at 4°C with gentle inversion. Agarose beads were collected by centrifugation at 4°C and 1000×g for 2 min and washed twice with 500 µl ice cold extraction buffer, followed by two washes with 1 ml RIPA buffer (50 mM Tris-HCL (pH 7.4), 150 mM NaCl, 1% NP-40, 0.25% Deoxycholate). Proteins were eluted from α -HA IgG-agarose beads by incubating 30 µl beads in 100 µl Laemmli sample buffer for 5 min at 95°C. 5 µl, 2.5 µl and 1.25 µl of samples (corresponding to 5%, 2.5% and 1.25% of eluates) were resolved by 10% SDS-PAGE and transferred to nitrocellulose membranes. Blots were probed with either 1∶20,000 α-HA antibodies (Roche) or 1∶2000 α-tubulin antibodies (Sigma #T9028, USA). Immunoreactive bands were visualized using horse radish peroxidase-conjugated secondary antibodies and detected via chemiluminescence (Amersham, USA). Band intensities were quantified using the Gel Analyzer tool in ImageJ (NIH).

### *In vitro* Acetyltransferase Assay

1, 2, 5, 10 or 20 µg of purified bovin e brain tubulin (Cytoskeleton Inc., USA) or 100 nM of phytic acid (Sigma #P5681, USA) was mixed with 2 µl of ^14^C-acetyl CoA (56 µCi/µM) in the absence or presence of 1 µg of HopZ1a, HopZ1a(C216A), or HopZ1a(K289R) in a 20 µl reaction containing 50 mM HEPES (pH 8.0), 10% glycerol, 1 mM DTT and was incubated for 1 hour at 30°C [15]. For Proteinase K-treated tubulin or phytic acid, 2 µg of tubulin or 100 nM of phytic acid was pre-incubated with 0.04 µg/µl (1/500) or 0.005 µg/µl (1/5000) of Proteinase K for 20 minutes at 37°C, and heat-inactivated for 10 minutes at 95°C. The reaction mixtures were loaded onto 12% SDS-PAGE gels and ran at 120 V for 90 minutes. The radioactive gels were fixed with 50% methanol and 10% glacial acetic acid for 30 minutes, followed by 15-minute incubation in the Amplify (Amersham, USA) enhancing solution. Gels were dried and placed in a phosphorimager cassette (Bio-Rad, USA) at −20°C for at least two weeks.

### Ion Leakage Assay

Leaves of four-week-old Arabidopsis Col-0 plants were syringe-infiltrated with *Pto*DC3000 at OD_600_ = 0.04 (∼2×10^7^ CFU/ml). Following inoculation, four leaf discs (1.5 cm^2^) were harvested from each plant, soaked in distilled water (dH_2_O) for 45 min, and transferred to 6 ml of dH_2_O. Conductivity readings were taken using an Orion 3 Star conductivity meter (Thermo Electron Corporation, USA).

### Bacterial Growth Assay

Arabidopsis *zar1-1* was syringe-infiltrated with *P. syringae* pv. cilantro 0788-9 (*Pci*0788-9) carrying *pUCP20*, *pUCP20-hopZ1a-HA* or *pUCP20-hopZ1a (K289R)-HA* at ∼1×10^5^ CFU/ml (OD600 = 0.0002). For the oryzalin experiments, Arabidopsis Col-0 or *zar1-1* plants were syringe-infiltrated with *Pto*DC3000, *P. syringae* pv. maculicola ES4326, *Pto*DC3000 carrying *pUCP20* or *pUCP20-hopZ1a-HA* in the presence of 1% ethanol or 100 µM oryzalin. Four leaf-discs (1 cm^2^) were collected from each plant and colony counts were performed on Day 0 and Day 3 using established methods [26]. The experiment was performed at least three times.

### Liquid Arabidopsis-*P. syringae* Infection Assay

Surface-sterilized *A. thaliana* GFP-MAP4 [37] and GFP-AtEB1 [38] seeds were grown as previously described [34]. Five-day-old seedlings were inoculated with *Pto*DC3000 carrying *pUCP20*, *pUCP20-hopZ1a-HA*, *pUCP20-hopZ1a(C216A)-HA*, or *pDSK519-avrRpt2* to a final concentration of 10^7^ CFU/mL for ∼16 hours.

### Confocal Microscopy

Epidermal layers of five- or six-day-old seedlings were visualized with a Leica DMI 6000B fluorescence microscope (Quorum Technologies, Canada). Z-stacks of 80 confocal images separated by 0.2 µm were taken and compressed into a 2D-image. Images were analyzed and quantified using the Volocity Quantification software (Quorum Technologies, Canada). For quantification of microtubules, regions in the images that correspond to guard cells were removed and fluorescence signals corresponding to microtubules from the pavement cells were quantified by Volocity Quantification module. The ends of microtubules were similarly quantified after removing regions in the images that correspond to guard cells.

### The secGFP Secretion Assay

HopZ1a-myc, HopZ1a(C216A)-myc, and AtSYP121-Sp2-myc were transiently expressed in leaves of *Nicotiana benthamiana* expressing secGFP using *Agrobacterium* infiltration. Extracellular (apoplastic) fluid was isolated from infiltrated leaves at 24 h post-inoculation using established protocols [41]. Intracellular proteins from the remaining leaf tissue were separated by SDS-PAGE followed by immunoblot analysis using an anti-GFP serum. To verify expression of the effector proteins, the blot was stripped and then probed with an anti-myc antibody.

### The *zar1-1/Dex:hopZ1a* Transgenic Plants

*zar1-1* plants [28] were transformed with *pBD::hopZ1a-HA* and *pBD::hopZ1a(C216A)-HA* [26] using the floral dip method. Transgenic plants were selected by Basta resistance and confirmed by PCR and sequencing. The *zar1-1* genotype was confirmed by PCR and by loss of the HopZ1a-induced hypersensitive response [28]. Homozygosity of T3 lines was determined by their segregation ratios on plates containing half-strength Murashige and Skoog (MS) media and 6 mg/L bialophos.

### Callose Staining and Image Analysis

Three-week-old *zar1-1* or *zar1-1/Dex:hopZ1a* transgenic line (2D) plants were sprayed with 30 µM dexamethasone to induce HopZ1a protein expression for 24 h. Leaves were then syringe-infiltrated with 10 µM of purified flg22 peptides and harvested 24 h post flg22-infiltration, cleared, and stained with 0.01% aniline blue for callose as previously described [47]. Leaves were visualized with a fluorescence microscope and the callose deposits were calculated using Image J software.

### Virulence Assay for Microtubule Destruction

100 µM of oryzalin (in 1% ethanol) or 1% ethanol alone was co-infiltrated with 1×10^5^ CFU/mL of *Pto*DC3000 or *Pto*DC3000 Δ*hrcC* into four-week old *A. thaliana* leaves. Four leaf-discs (1 cm^2^) were collected from each plant and colony counts were performed on Day 0 and Day 3 following standard growth assay methods [26].

## Supporting Information

Figure S1 **HopZ1a binds unassembled tubulin heterodimers.** (**A**) Recombinant HopZ1a, HopZ1a(C216A) and BSA were immobilized on the surface of a Biacore CM5 sensor chip at the following response units (RU), 16386 RU, 12808 RU and 21941 RU, respectively. 250 µg/ml of bovine brain tubulin was flowed across the recombinant HopZ1a, HopZ1a(C216A) and BSA -bound surface, generating a RU difference of 630 RU, 463 RU and −1.6 RU, respectively. (**B**) 4108RU of GST-HopZ1a and 3036RU of GST were immobilized on the surface of a Biacore CM5 sensor chip. 500 µg/ml of bovine brain tubulin was flowed across the GST-HopZ1a and GST -bound surface, generating a RU difference of 36 RU and −2.8 RU, respectively. (**C**) HIS-HopZ1a, GST-HopZ1a and GST were immobilized on the surface of a Biacore CM5 sensor chip at the following RU: 6953 RU, 8392 RU and 9315 RU, respectively. 500 µg/ml of soybean tubulin was flowed across the HIS-HopZ1a, GST-HopZ1a and GST -bound surface, generating a RU difference of 1233 RU, 305 RU and −135 RU, respectively.
(TIF)

Figure S2 **HIS-HopF2 does not bind microtubules.** Immunoblot analysis of HIS-HopZ1a and HIS-HopF2 in a microtubule co-sedimentation assay detected with rabbit α-HIS antibody. In the absence of microtubules, HIS-HopZ1a and HIS-HopF2 proteins were found only in the supernatant (S) fractions. In the presence of microtubules, HIS-HopZ1a proteins were found predominantly in the pellet (P) fraction, while HIS-HopF2 proteins were found predominantly in the supernatant (S) fraction.
(TIF)

Figure S3 **The acetyltransferase activity of HopZ1a is activated by phytic acid, which results in HopZ1a autoacetylation** ***in cis*** **and acetylation of tubulin.** (**A**) Purified recombinant GST-HopZ1a or HIS-HopZ1a proteins were incubated with or without 20 µg of tubulin heterodimers or 20 µg of BSA in the presence of ^14^C-labeled acetyl-CoA for 1 hour at 30°C. BSA did not activate the acetyltransferase activity of HopZ1a. (**B**) Purified GST-HopZ1a proteins were incubated with 1 µg, 2 µg, 5 µg, or 10 µg of tubulin heterodimers in the presence of ^14^C-labeled acetyl-CoA for 1 hour at 30°C. GST-HopZ1a acetylated tubulin at ∼1∶1 molar ratio (1 µg of GST-HopZ1a to 2 µg of tubulin). (**C**) Purified GST-HopZ1a proteins were incubated with 100 nM of phytic acid alone, 2 µg of tubulin pre-treated with Proteinase K or 100 nM of phytic acid pre-treated with Proteinase K, in the presence of ^14^C-labeled acetyl-CoA for 1 hour at 30°C, The contaminating phytic acid in tubulin activates HopZ1a's acetyltransferase activity (**D**) Purified HIS-HopZ1a (∼42 kDa) and GST-HopZ1a (C216A) (∼68 kDa) proteins were incubated with or without 10 µg of tubulin heterodimers or 100 nM phytic acid in the presence of ^14^C-labeled acetyl-CoA for 1 hour at 30°C. HopZ1a autoacetylates *in cis*. All samples were separated by 12% SDS-PAGE and the ^14^C-incorporation was analyzed by Phosphorimager.
(TIF)

Figure S4 **HopZ1a(K289R) binds tubulin heterodimers and does not have basal autoacetylation activity.** (**A**) 5634RU of HIS-HopZ1a and 5589RU of HIS-HopZ1a(K289R) were immobilized on the surface of a Biacore CM5 sensor chip. 500 µg/ml of bovine brain tubulin was flowed across the HIS-HopZ1a and HIS-HopZ1a(K289R) -bound surface, generating a RU difference of 117RU and 186RU, respectively. (**B**) Purified recombinant GST-HopZ1a, GST-HopZ1a(C216A) and GST-HopZ1a (K289R) proteins were incubated in the presence of ^14^C-labeled acetyl-CoA for 1 hour at 30°C. All samples were separated by 12% SDS-PAGE and the ^14^C-incorporation was analyzed by Phosphorimager.
(TIF)

Figure S5 **HopZ1a does not affect the actin cytoskeleton.** Confocal microscopy images of five-day-old GFP-Talin seedlings infected with *Pto*DC3000 expressing empty vector *pUCP20*, *pUCP20-hopZ1a-HA*, or *pUCP20-hopZ1a(C216A)-HA* for ∼16 hours. Scale bar = 25 µm.
(TIF)

Figure S6 **Microtubule destruction phenocopies HopZ1a virulence activity.** *P. syringae* growth assay in Arabidopsis *zar1-1*. In the presence of microtubule inhibitor, oryzalin, *Pto*DC3000 (DC) grew significantly better than DC alone after three days, The growth of *P. syringae* carrying *pUCP20-hopZ1a* [DC(HopZ1a)] is not affected by the presence or absence of oryzalin. Experiments were repeated two times and the data from one representative experiment is presented. [(*) indicate statistical significance. P<0.05, two-tailed t-test.]
(TIF)

Figure S7 **HopZ1a inhibits cell wall-based defense.** (**A**) *zar1-1* and *zar1-1/Dex:hopZ1a* transgenic leaves were sprayed with water (−DEX) or 30 µM dexamethasone to induce HopZ1a protein expression (+DEX) for 24 h. Leaves were then syringe-infiltrated with 10 µM of flg22 for 24 h, followed by clearing and staining with 0.01% Aniline blue for callose. Expression of HopZ1a (+DEX) suppressed flg22-induced callose deposition. (**B**) Quantification of callose depositions of 16 images per treatment. Error bars indicate standard error.
(TIF)

Table S1 **Peptides identified by LC-MS/MS analysis from representative TAP experiments.** (**A**) HopZ1a and (**B**) Radil [67] expressed in HEK293T cells.
(TIF)
