# Supplementary figures and images for: A Bacterial Acetyltransferase Destroys Plant Microtubule Networks and Blocks Secretion
Source: PLoS Pathog. 2012 Feb 2;8(2):e1002523. doi: 10.1371/journal.ppat.1002523 (PMC3271077; doi:10.1371/journal.ppat.1002523)

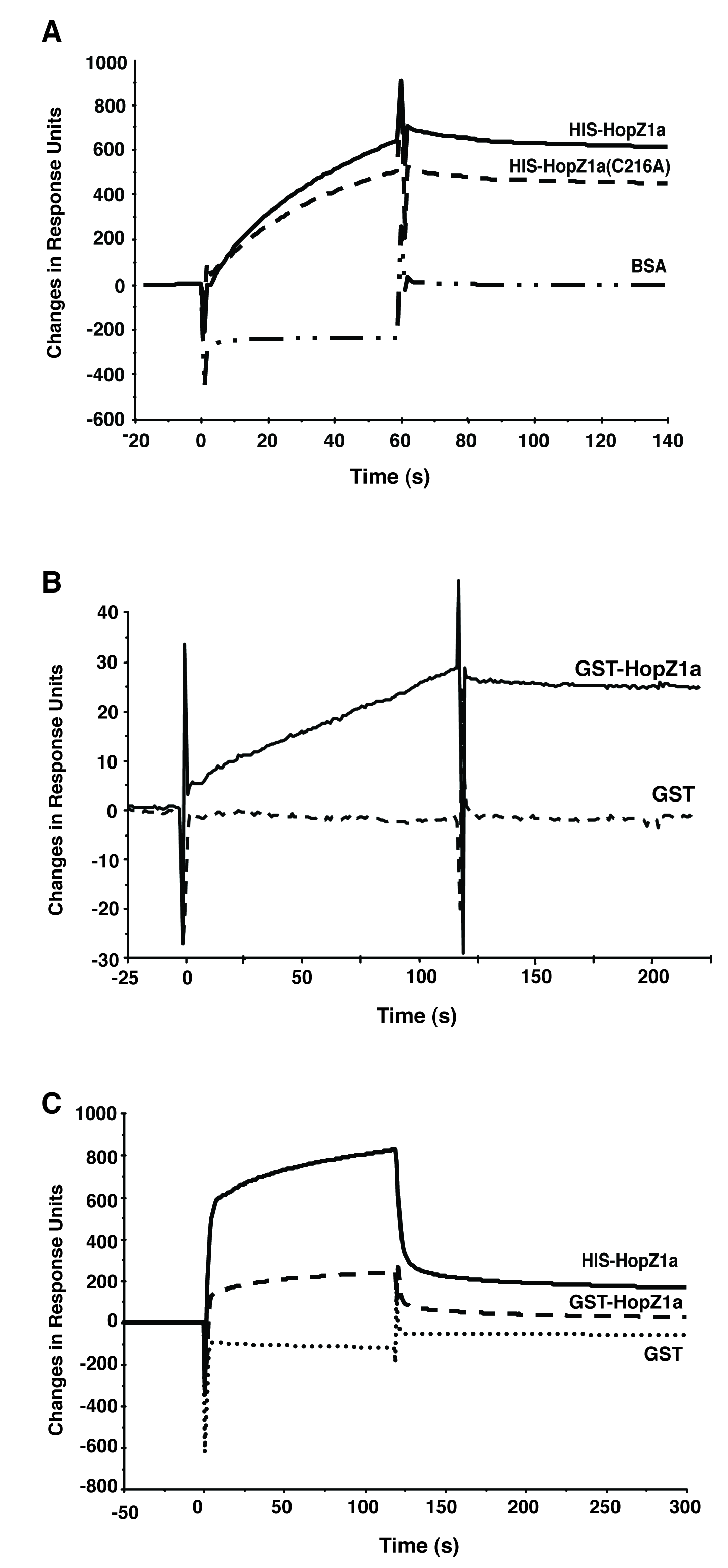

Supplement: Figure S1 — HopZ1a binds unassembled tubulin heterodimers. (A) Recombinant HopZ1a, HopZ1a(C216A) and BSA were immobilized on the surface of a Biacore CM5 sensor chip at the following response units (RU), 16386 RU, 12808 RU and 21941 RU, respectively. 250 µg/ml of bovine brain tubulin was flowed across the recombinant HopZ1a, HopZ1a(C216A) and BSA -bound surface, generating a RU difference of 630 RU, 463 RU and −1.6 RU, respectively. (B) 4108RU of GST-HopZ1a and 3036RU of GST were immobilized on the surface of a Biacore CM5 sensor chip. 500 µg/ml of bovine brain tubulin was flowed across the GST-HopZ1a and GST -bound surface, generating a RU difference of 36 RU and −2.8 RU, respectively. (C) HIS-HopZ1a, GST-HopZ1a and GST were immobilized on the surface of a Biacore CM5 sensor chip at the following RU: 6953 RU, 8392 RU and 9315 RU, respectively. 500 µg/ml of soybean tubulin was flowed across the HIS-HopZ1a, GST-HopZ1a and GST -bound surface, generating a RU difference of 1233 RU, 305 RU and −135 RU, respectively. (TIF) [file ppat.1002523.s001.tif]

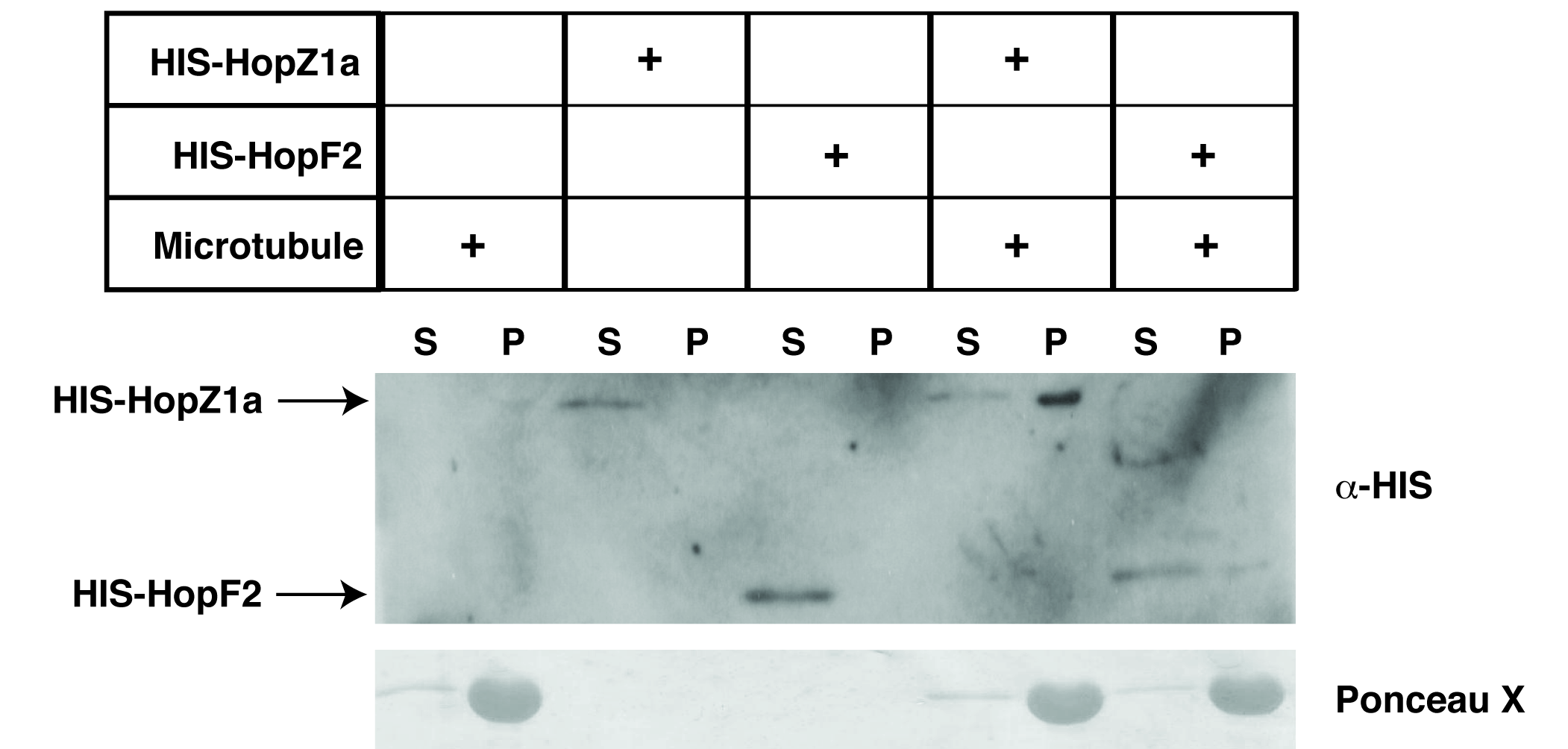

Supplement: Figure S2 — HIS-HopF2 does not bind microtubules. Immunoblot analysis of HIS-HopZ1a and HIS-HopF2 in a microtubule co-sedimentation assay detected with rabbit α-HIS antibody. In the absence of microtubules, HIS-HopZ1a and HIS-HopF2 proteins were found only in the supernatant (S) fractions. In the presence of microtubules, HIS-HopZ1a proteins were found predominantly in the pellet (P) fraction, while HIS-HopF2 proteins were found predominantly in the supernatant (S) fraction. (TIF) [file ppat.1002523.s002.tif]

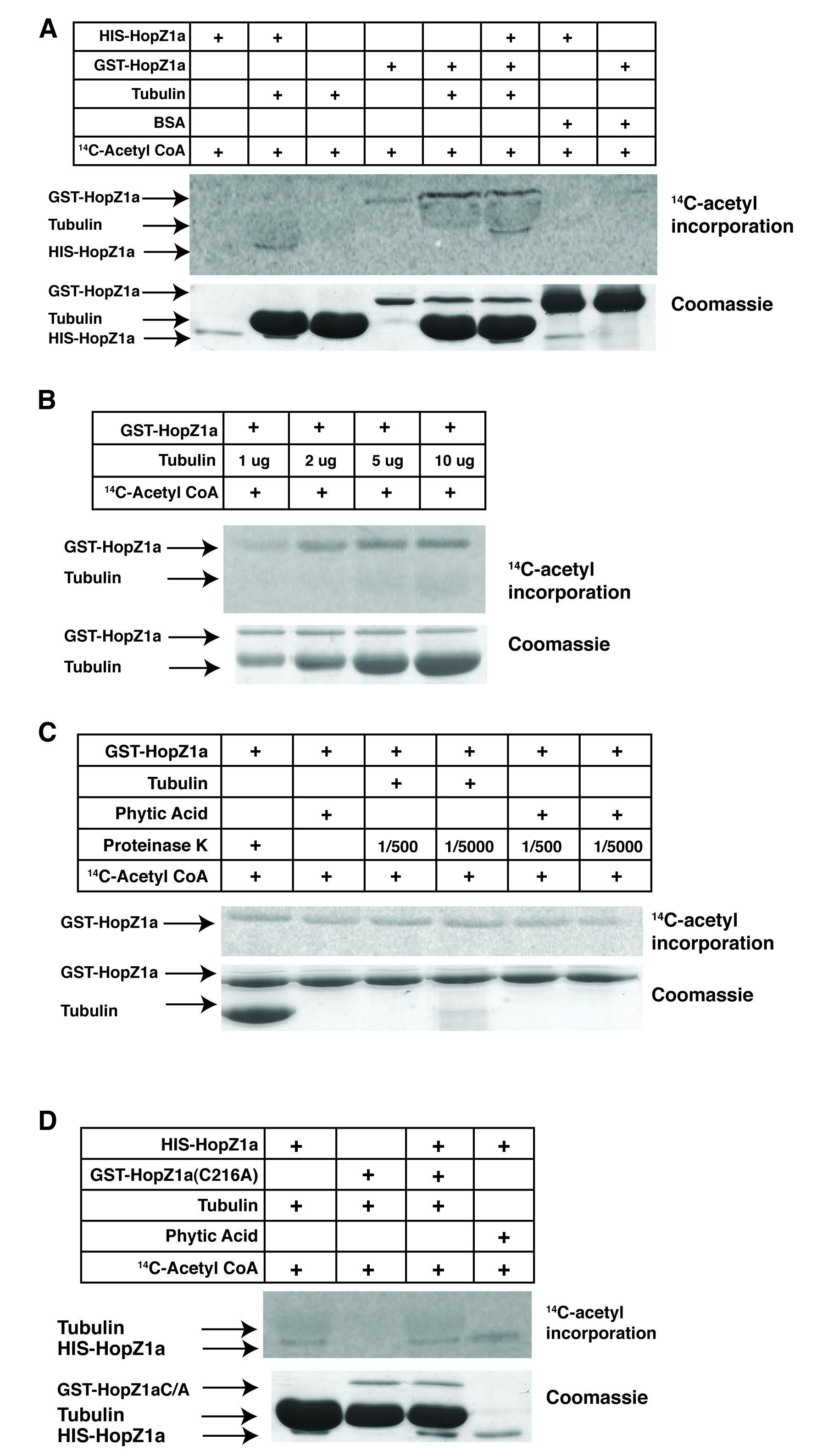

Supplement: Figure S3 — The acetyltransferase activity of HopZ1a is activated by phytic acid, which results in HopZ1a autoacetylation in cis and acetylation of tubulin. (A) Purified recombinant GST-HopZ1a or HIS-HopZ1a proteins were incubated with or without 20 µg of tubulin heterodimers or 20 µg of BSA in the presence of 14C-labeled acetyl-CoA for 1 hour at 30°C. BSA did not activate the acetyltransferase activity of HopZ1a. (B) Purified GST-HopZ1a proteins were incubated with 1 µg, 2 µg, 5 µg, or 10 µg of tubulin heterodimers in the presence of 14C-labeled acetyl-CoA for 1 hour at 30°C. GST-HopZ1a acetylated tubulin at ∼1∶1 molar ratio (1 µg of GST-HopZ1a to 2 µg of tubulin). (C) Purified GST-HopZ1a proteins were incubated with 100 nM of phytic acid alone, 2 µg of tubulin pre-treated with Proteinase K or 100 nM of phytic acid pre-treated with Proteinase K, in the presence of 14C-labeled acetyl-CoA for 1 hour at 30°C, The contaminating phytic acid in tubulin activates HopZ1a's acetyltransferase activity (D) Purified HIS-HopZ1a (∼42 kDa) and GST-HopZ1a (C216A) (∼68 kDa) proteins were incubated with or without 10 µg of tubulin heterodimers or 100 nM phytic acid in the presence of 14C-labeled acetyl-CoA for 1 hour at 30°C. HopZ1a autoacetylates in cis. All samples were separated by 12% SDS-PAGE and the 14C-incorporation was analyzed by Phosphorimager. (TIF) [file ppat.1002523.s003.tif]

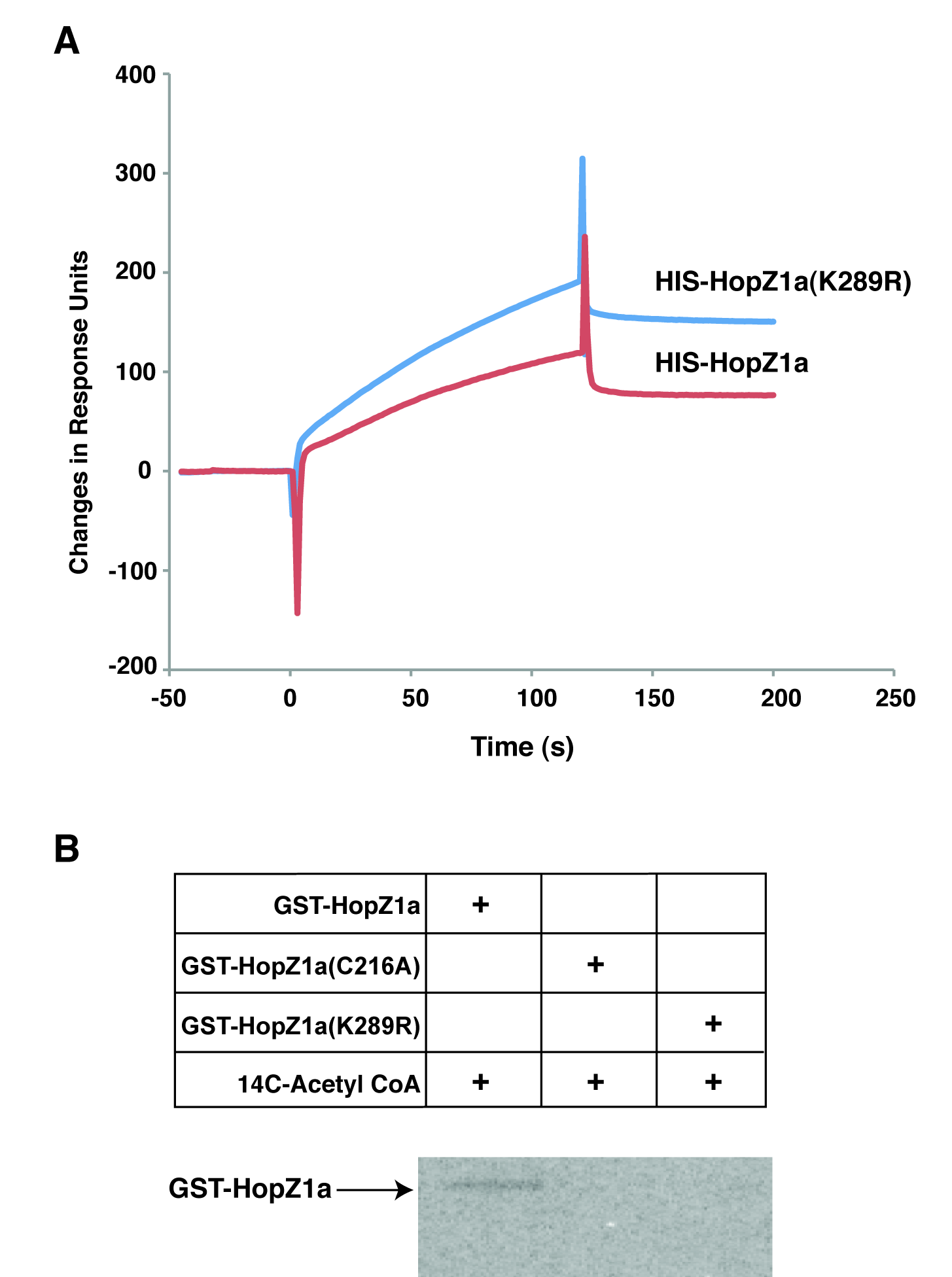

Supplement: Figure S4 — HopZ1a(K289R) binds tubulin heterodimers and does not have basal autoacetylation activity. (A) 5634RU of HIS-HopZ1a and 5589RU of HIS-HopZ1a(K289R) were immobilized on the surface of a Biacore CM5 sensor chip. 500 µg/ml of bovine brain tubulin was flowed across the HIS-HopZ1a and HIS-HopZ1a(K289R) -bound surface, generating a RU difference of 117RU and 186RU, respectively. (B) Purified recombinant GST-HopZ1a, GST-HopZ1a(C216A) and GST-HopZ1a (K289R) proteins were incubated in the presence of 14C-labeled acetyl-CoA for 1 hour at 30°C. All samples were separated by 12% SDS-PAGE and the 14C-incorporation was analyzed by Phosphorimager. (TIF) [file ppat.1002523.s004.tif]

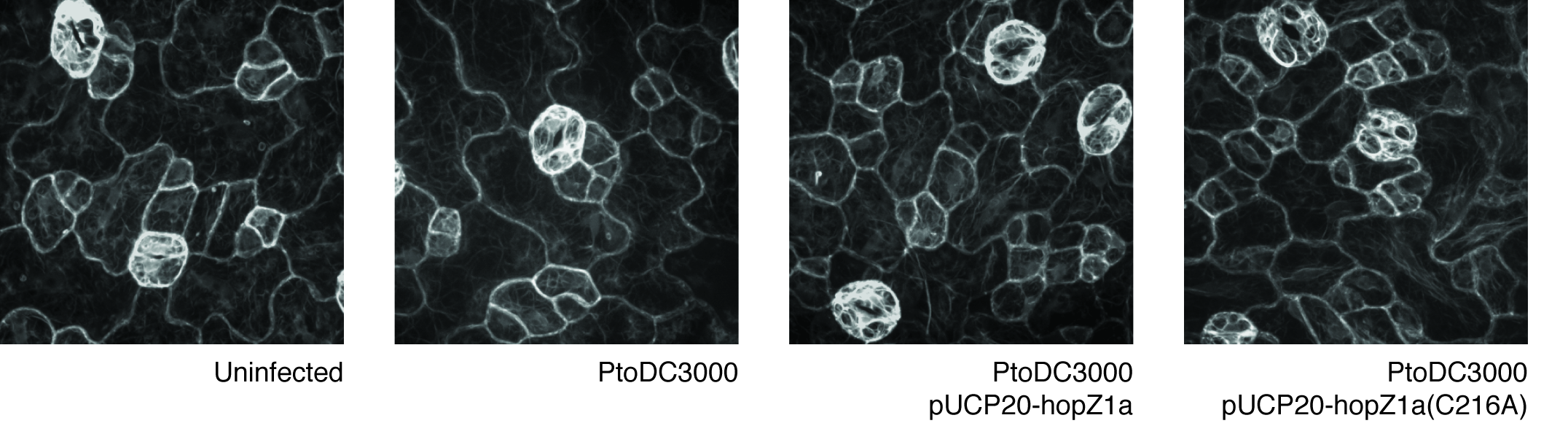

Supplement: Figure S5 — HopZ1a does not affect the actin cytoskeleton. Confocal microscopy images of five-day-old GFP-Talin seedlings infected with PtoDC3000 expressing empty vector pUCP20, pUCP20-hopZ1a-HA, or pUCP20-hopZ1a(C216A)-HA for ∼16 hours. Scale bar = 25 µm. (TIF) [file ppat.1002523.s005.tif]

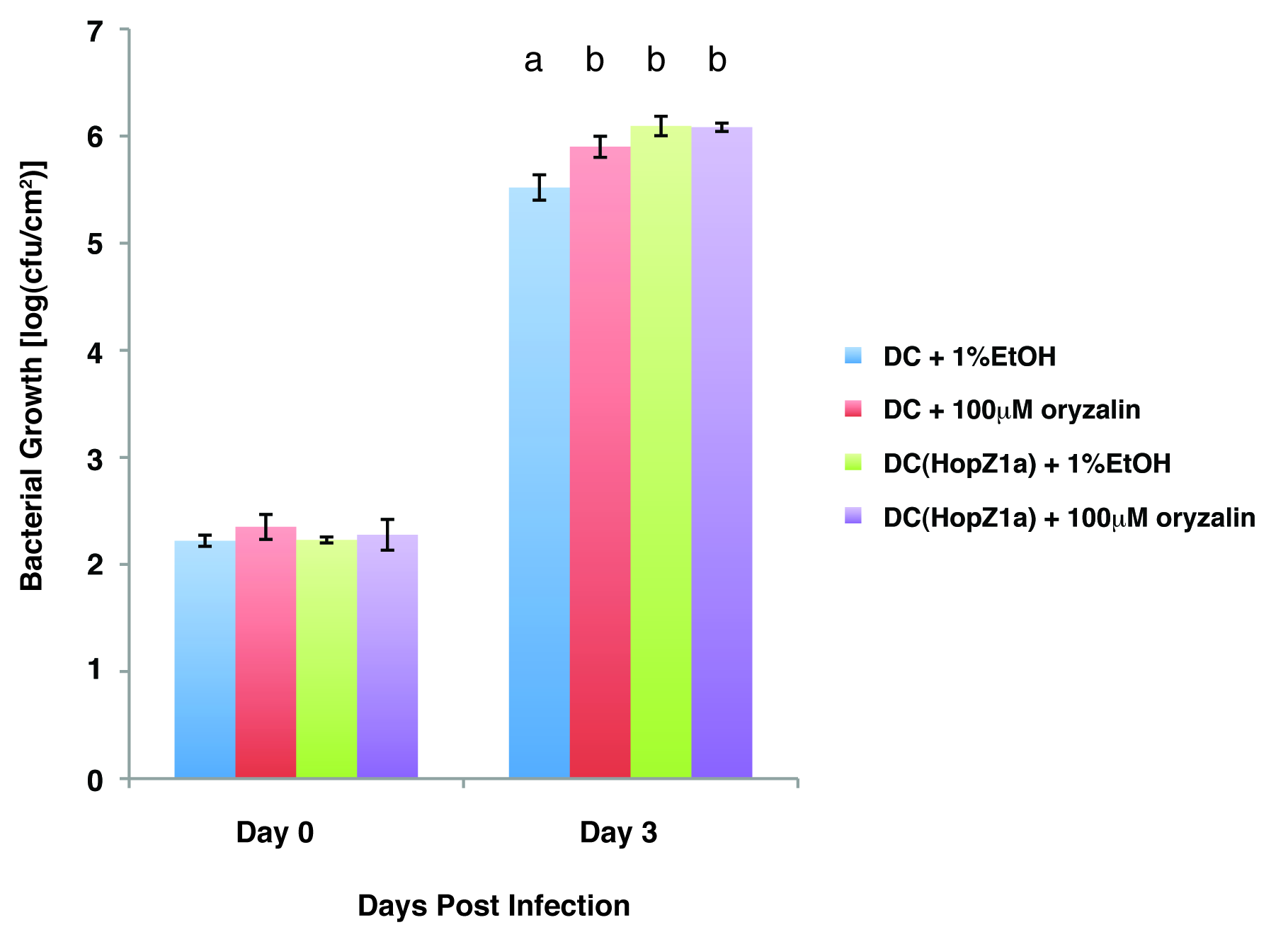

Supplement: Figure S6 — Microtubule destruction phenocopies HopZ1a virulence activity. P. syringae growth assay in Arabidopsis zar1-1. In the presence of microtubule inhibitor, oryzalin, PtoDC3000 (DC) grew significantly better than DC alone after three days, The growth of P. syringae carrying pUCP20-hopZ1a [DC(HopZ1a)] is not affected by the presence or absence of oryzalin. Experiments were repeated two times and the data from one representative experiment is presented. [(*) indicate statistical significance. P<0.05, two-tailed t-test.] (TIF) [file ppat.1002523.s006.tif]

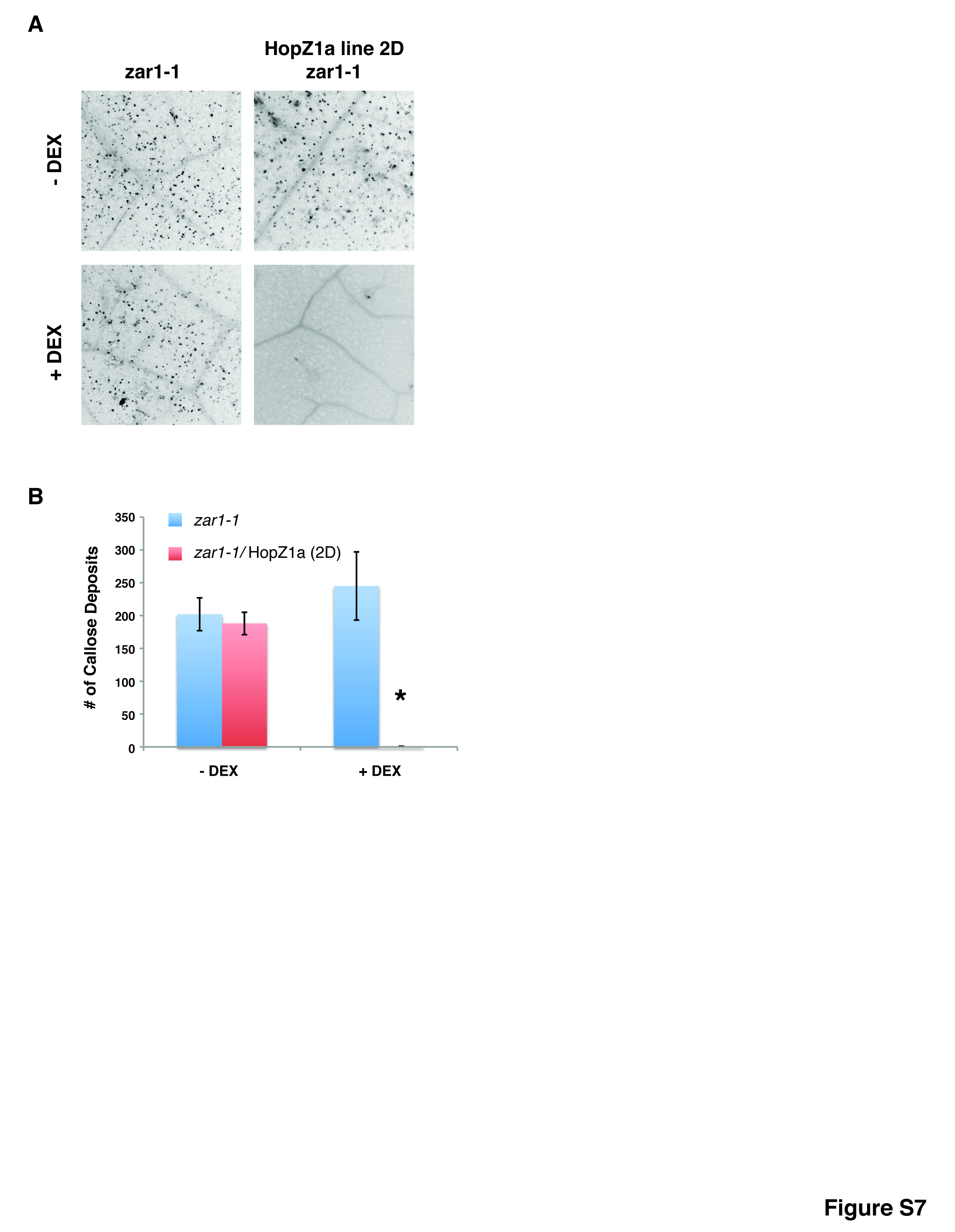

Supplement: Figure S7 — HopZ1a inhibits cell wall-based defense. (A) zar1-1 and zar1-1/Dex:hopZ1a transgenic leaves were sprayed with water (−DEX) or 30 µM dexamethasone to induce HopZ1a protein expression (+DEX) for 24 h. Leaves were then syringe-infiltrated with 10 µM of flg22 for 24 h, followed by clearing and staining with 0.01% Aniline blue for callose. Expression of HopZ1a (+DEX) suppressed flg22-induced callose deposition. (B) Quantification of callose depositions of 16 images per treatment. Error bars indicate standard error. (TIF) [file ppat.1002523.s007.tif]

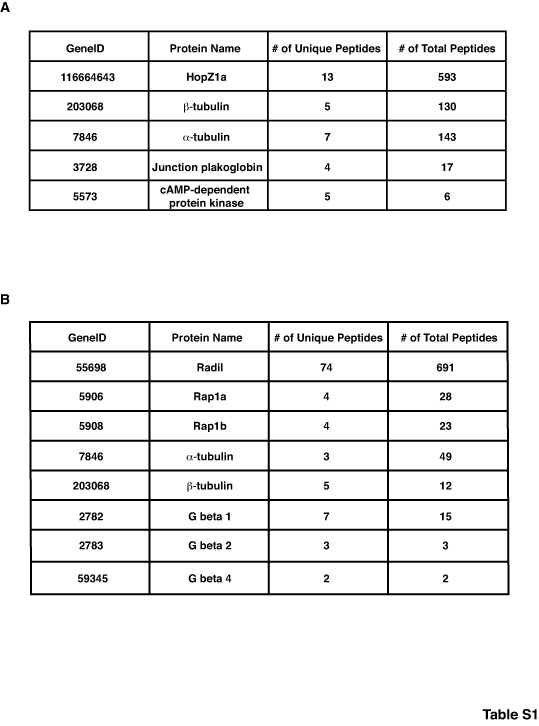

Supplement: Table S1 — Peptides identified by LC-MS/MS analysis from representative TAP experiments. (A) HopZ1a and (B) Radil [67] expressed in HEK293T cells. (TIF) [file ppat.1002523.s008.tif]
